# Rational Mitomycin Nanocarriers Based on Hydrophobically Functionalized Polyelectrolytes and Poly(lactide-*co*-glycolide)

**DOI:** 10.1021/acs.langmuir.1c03360

**Published:** 2022-04-20

**Authors:** Łukasz Lamch, Kazimiera A. Wilk, Imre Dékány, Ágota Deák, Viktória Hornok, László Janovák

**Affiliations:** † Department of Engineering and Technology of Chemical Processes, Faculty of Chemistry, Wrocław University of Science and Technology, Wybrzeże Wyspiańskiego 27, Wrocław 50-370, Poland; ‡ Department of Physical Chemistry and Materials Science, University of Szeged, Rerrich Béla tér 1, Szeged H-6720, Hungary

## Abstract

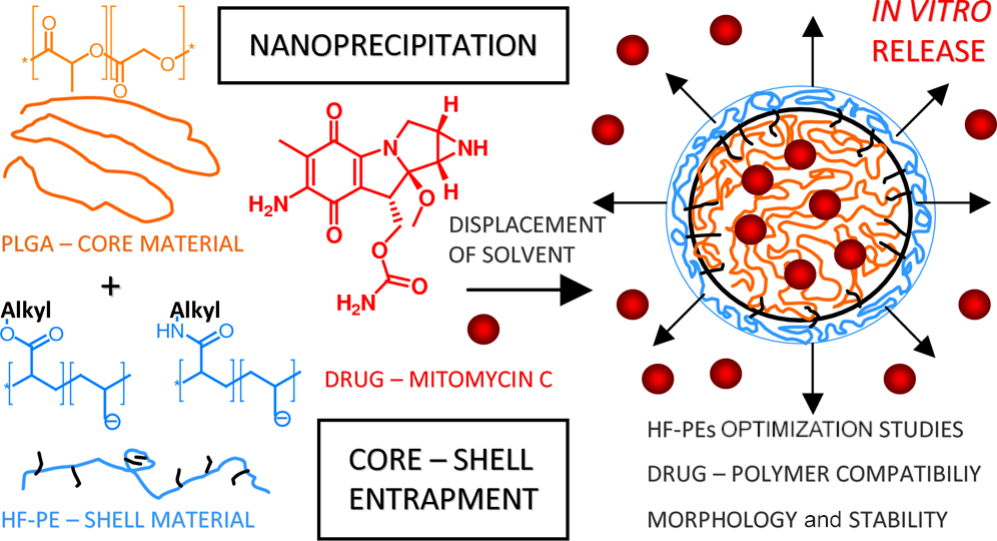

Encapsulation of hydrophilic and amphiphilic drugs in appropriate colloidal carrier systems for sustained release is an emerging problem. In general, hydrophobic bioactive substances tend to accumulate in water-immiscible polymeric domains, and the release process is controlled by their low aqueous solubility and limited diffusion from the nanocarrier matrix. Conversely, hydrophilic/amphiphilic drugs are typically water-soluble and insoluble in numerous polymers. Therefore, a core–shell approach—nanocarriers comprising an internal core and external shell microenvironments of different properties—can be exploited for hydrophilic/amphiphilic drugs. To produce colloidally stable poly(lactic-*co*-glycolic) (PLGA) nanoparticles for mitomycin C (MMC) delivery and controlled release, a unique class of amphiphilic polymers—hydrophobically functionalized polyelectrolytes—were utilized as shell-forming materials, comprising both stabilization via electrostatic repulsive forces and anchoring to the core via hydrophobic interactions. Undoubtedly, the use of these polymeric building blocks for the core–shell approach contributes to the enhancement of the payload chemical stability and sustained release profiles. The studied nanoparticles were prepared via nanoprecipitation of the PLGA polymer and were dissolved in acetone as a good solvent and in an aqueous solution containing hydrophobically functionalized poly(4-styrenesulfonic-*co*-maleic acid) and poly(acrylic acid) of differing hydrophilic–lipophilic balance values. The type of the hydrophobically functionalized polyelectrolyte (HF-PE) was crucial for the chemical stability of the payload—derivatives of poly(acrylic acid) were found to cause very rapid degradation (hydrolysis) of MMC, in contrast to poly(4-styrenesulfonic-*co*-maleic acid). The present contribution allowed us to gain crucial information about novel colloidal nanocarrier systems for MMC delivery, especially in the fields of optimal HF-PE concentrations, appropriate core and shell building materials, and the colloidal and chemical stability of the system.

## Introduction

Encapsulation of bioactive hydrophilic or amphiphilic compounds, that is, possessing both lipophilic and lipophobic moieties, is an emerging problem. The aforementioned molecules are very often water-soluble, making it difficult to prepare stable dispersions in aqueous systems; moreover, in contrast to hydrophobic drugs and chemotherapeutics, they are more susceptible to chemical or enzymatic degradation, for example, hydrolysis, leading to the loss of appropriate activity or even the formation of toxic byproducts.^1,2^ Therefore, an appropriate hydrophilic/amphiphilic nanocarrier should consist of at least two different microenvironments of the utmost character to provide both chemical stability of the drug and a sustained release profile. The aforementioned requirements are fulfilled for self-assembled structures, such as polymeric micelles, polymersomes, and liposomes,^2−4^ as well as core–shell nanocarriers composed (generally) of hydrophobic internal cores stabilized by outer amphiphilic shell layers.^1,5,6,7^ The most common methods for the so-called core–shell encapsulation are both physical (self-assembly in an appropriate selective solvent and nanoprecipitation) and chemical (polymerization of amphiphilic monomers or oligomers and cross-linking of self-assembled core–shell structures).^2,8^ Nanoprecipitation (solvent displacement and interfacial deposition—cosolvent removal method) consists of a facile, mild, and low-energy process characterized by excellent reproducibility and high potential for upscaling toward industrial applications.^9^ The most important principle of this approach is the spontaneous emulsification of the organic internal phase with the aqueous external part.^8,10^ In general, the process of nanoprecipitation comprises nucleation, growth, and aggregation, in which the driving force is supersaturation.^11^ The nucleation rate is dependent on the mixing of the aqueous and organic phases, leading to local supersaturation of the polymer.^12^ Generally, the aqueous phase (a liquid characterized by high surface tension) tends to attract the surroundings more strongly than an organic solvent (liquid of a low surface tension). The diffusion of solvent molecules occurs from the bulk organic phase (i.e., the area with low surface tension), causing gradual precipitation, followed by the formation of core–shell structures.^13^ Conversely, a similar process can be driven by ultralow interfacial tension (i.e., when the difference between the surface tensions of the two liquids is close to zero or negligible) when an appropriate amphiphilic substance is added to the aqueous phase. The gradual replacement of the solvent between phases leads to the formation of core–shell nanoparticles with internal subdomains composed of organic solvent-soluble polymers and outer layers of water-soluble materials (e.g., surfactants or polymers).^1,2,8^ The aforementioned processes depend primarily on the concentrations of both water and organic solvent-borne materials, the rate of addition, and the speed of mixing.^8^ A unique feature of nanoprecipitation is the opportunity to adjust the size and polydispersity of core–shell nanoparticles by optimizing the concentration of polymers, their hydrophobicity molecular weight, and the process conditions.^12^

The design of an appropriate carrier for hydrophobic/amphiphilic bioactive compounds should consider the following points of interest: (i) the biodegradability, after fulfilling their purposes, and biocompatibility of particular building blocks; (ii) compatibility between particular building blocks and the payload; (iii) the desired hydrodynamic diameters of nanocarriers; (iv) the type and concentration of the amphiphilic stabilizing shell material; and (v) the stability of the payload in carrier microenvironments.^1,2,8,14^ A very important group of building blocks for nanocarriers consists of biodegradable, synthetic polyesters, such as polycaprolactone (PCL), polylactide (PLA) and its tactic stereoisomers (poly l-lactide , poly d-lactide, and poly d,l-lactide), poly(lactide-*co*-glycolide) (PLGA), and polyhydroxybutyrate. These polymers and their derivatives, such as block copolymers with poly(ethylene oxide) chains, have been extensively studied for biomedical applications.^15,16^ In contrast to biopolymer synthesis, further modification/functionalization of the aforementioned polyesters is easily tunable, especially in their mean molecular weight and their distribution, as well as ending groups. It should be noted that due to the structure comprising repeating units of shorter or longer hydrocarbon chains separated by ester bonds, these could be compatible with hydrophilic, hydrophobic, and amphiphilic payloads, while products of their biodegradation/hydrolysis constitute nontoxic hydroxycarboxylic acids.^2,17,18^ Good compatibility with different bioactive compounds makes synthetic polyesters good candidates for nanocarrier preparation. It should be noted that the properties of polyesters, including their mean molecular weight, hydrophobicity, and drug loading capacity, can be tuned via modification of the synthesis conditions.^17^ One of the most important drawbacks of synthetic polyester usage as nanocarriers is their colloidal instability in aqueous systems—due to their complete aqueous insolubility, their water dispersions tend to aggregate and undergo sedimentation.^8^ To prevent the aforementioned unwanted processes, synthetic polyesters can be either chemically bonded to hydrophilic polymers [especially poly(ethylene oxide)] or nanoprecipitated via water into colloidally stable core–shell type dispersions.^1,8^ The first approach—preparation of polymeric micelles/nanoparticles of block copolymers—has numerous drawbacks, including poor control over their sizes, which are mostly dependent on the length of particular polymeric blocks. Core–shell nanoprecipitation allows for obtaining both electrically charged (i.e., stabilized by ionic surfactants or polyelectrolytes) and neutral (i.e., using a shell layer built of noncharged polymers or other amphiphilic substances) controlled dimensions and morphology.^19^

The design of our system, that is, PLGA nanoparticles stabilized by hydrophobically functionalized polyelectrolytes (HF-PEs) as an efficient delivery system for mitomycin C (MMC), comprised the use of biocompatible and biodegradable building blocks. Traditional stabilizing materials for nanoparticles, that is, surfactants, polyelectrolytes and water-soluble polymers, might not be sufficiently stably anchored to the surfaces of nanoparticles, leading to fast desorption, followed by nanoparticle agglomeration and sedimentation.^19−22^ Therefore, our nanocarrier system is stabilized by a novel class of amphiphiles: hydrophilically functionalized polyelectrolytes with alkyl side chains attached to the backbone via labile chemical bonds, combining the advantages of both surfactants (amphiphilic structure and easy and stable adsorption on interfaces) and polyelectrolytes (stabilization by strong electrostatic and repulsive forces and low dependence on concentration changes or dilution).^16,20^ It should be emphasized that labile linking groups (ester or amide) between hydrophobic side chains and the polyelectrolyte backbone allow for biodegradation or chemical hydrolysis of the aforementioned compound, leading to the loss of its amphipilic character, thus reducing the risk of harmful behavior during systemic circulation. Moreover, in contrast to nonhydrophobically functionalized polyelectrolytes, as well as low-molecular-weight surfactants, our derivatives might effectively stabilize nanoparticles even at very low concentrations.^23,24^

We used MMC as a model drug during this study. MMC is an antineoplastic, antifibrotic,^25^ and antibiotic moderately water-soluble antiproliferative bifunctional alkylating agent that inhibits DNA synthesis by bonding to DNA chains.^26^ This chemotherapeutic is widely used for the treatment of solid tumors, such as carcinomas of the breast, esophagus, cervix, and bladder.^27^ MMC administered in a free form (e.g., subcutaneous injection) is excreted from the body in a very short period of time.^28^ Therefore, to provide the prolonged therapeutic effect of the drug, it is necessary to design and apply drug delivery systems that are capable of sustained drug release.

Hydrophobically functionalized derivatives of poly(acrylic acid) (PAA) and poly(4-styrenesulfonic-*co*-maleic acid) comprise a novel group of amphiphilic polymers with a negatively charged, water-soluble backbone and alkyl side chains, bearing an increment of hydrophobic character. Such a structure comprises a unique possibility of combining properties connected with the polymeric character (long backbone) and surfactant-like building blocks (amphiphilic units within one macromolecule) as well as high potential for hydrophilic–lipophilic balance (HLB) modification by changing the grafting ratio or alkyl side chain length. Therefore, in order to compare different HF-PEs, we have calculated HLB values for the studied amphiphiles using the conventional Davis expanded scale (utilizing positive or negative increments of particular chemical motifs) and the universal McGowan scale, allowing calculation based only on the averaged number of particular atoms and bonds within the chemical structure. It should be noted that such an approach is not limited only to low-molecular-weight surfactants but may be also utilized for a very broad group of organic compounds, when their amphiphilic character is negligible or their mean molecular weight is high. Moreover, it should be noted that the choice of backbone-forming polyelectrolytes is supported by numerous studies on their usefulness toward biologically active compound carrier systems.

**Scheme 1:**
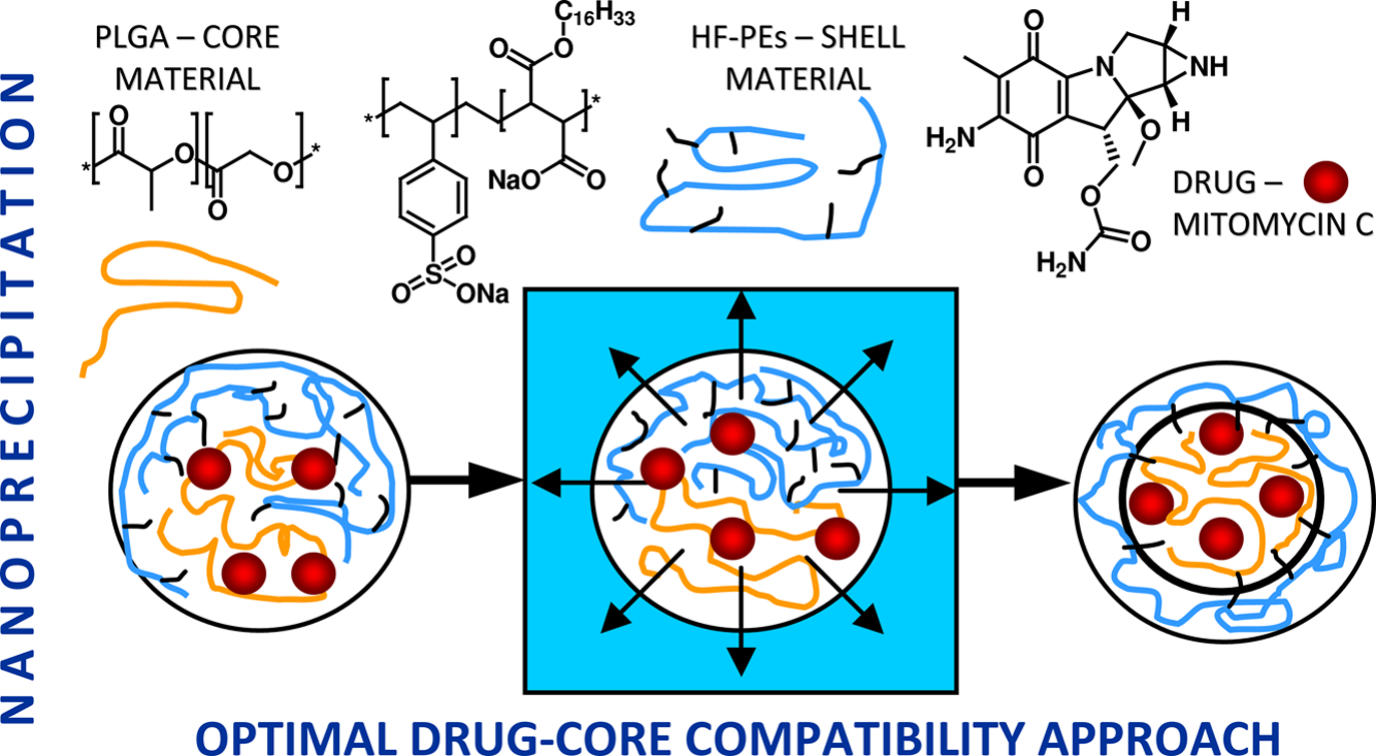
Graphical Representation of the Performed Studies

The aim of this work was to design, prepare, and carefully study the usefulness of PLGA nanoparticles stabilized by HF-PEs of different hydrophobicities and structures as nanocarriers for MMC delivery. The major points of interest were optimization of HF-PEs [namely, PSS-MA-*g*-C_16_OH (15%), PSS-MA-*g*-C_16_NH_2_ (15%), PAA-*g*-C_16_OH (15%), or PAA-*g*-C_12_OH (40%)—see Table 1 for their structures and characteristics] concentrations, colloidal stability [assessed using dynamic light scattering (DLS) and zeta potential measurements], and morphology [scanning electron microscopy (SEM) and transmission electron microscopy (TEM)] of the obtained nanocarriers, and chemical stability of the payload in carrier system microenvironments and its sustained release (UV–vis spectrometry). To systematically describe and investigate the solubility parameter (δ) of the studied systems, including their dispersion forces (δ_d_), polar forces (δ_p_), and hydrogen bonding (δ_h_) increments, the Flory–Huggins interaction parameter (χ) and kinetic constants for release profiles were assessed according to different models and compared with experimental properties. Our complex studies showed the usefulness of the core–shell approach for MMC delivery and indicated the most crucial points of interest for colloidal and chemical systems. The main aim of the study is shown in Scheme 1.

**Table 1:**
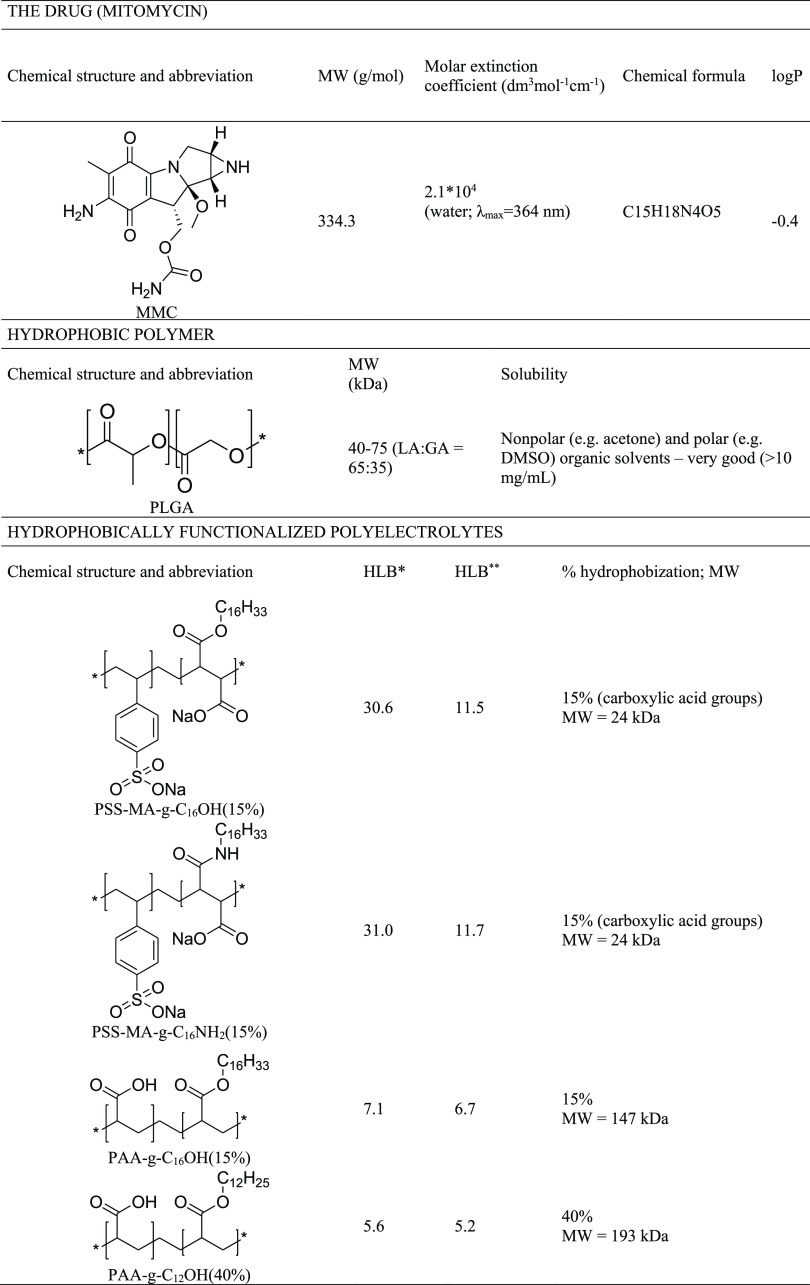
Structures and Properties of the Studied Hydrophobically Modified Polyelectrolytes, Hydrophobic Polymer (PLGA), and Drug (MMC)

a Calculated according to the Davis expanded scale.^29^

b Calculated according to the universal McGowan scale.^30^

## Experimental Section

### Materials

All of the reagents were used as received. PLGA (*M*_w_ = 45–70 kDa; LA/GA = 65:35), poly(4-styrenesulfonic-*co*-maleic acid) sodium salt (PSS/MA) (*M*_w_ = 20 kDa, styrenesulfonic acid/maleic acid = 1:1), PAA (*M*_w_ = 100 kDa, 35 wt % in H_2_O), and coupling agents [*N*,*N*′-dicyclohexylcarbodiimide (DCC), 4-*N*,*N*′-dimethylaminopyridine (DMAP), and *N*-hydroxysuccinimide (NHS)] were of reagent grade and obtained from Sigma-Aldrich (Burlington, MA, USA). Fatty alcohols (dodecanol and hexadecanol) and hexadecylamine were all of reagent grade (purity > 96%) and obtained from Fluka (Morristown, NJ, USA). All of the solvents used were of reagent or analytical grade and purchased from Avantor Performance Materials (Gliwice, Poland) or Molar Chemicals Kft (Halásztelek, Hungary). The water used in all of the experiments was doubly distilled and purified by means of a Millipore (Bedford, MA, USA) Milli-Q purification system.

### Synthesis of Hydrophobically Functionalized Poly(styrenesulfonic-*co*-maleic Acid)

Hydrophobically (hexadecyl side chains, 15% nominal degree of hydrophobization per carboxylic acid group) functionalized poly(4-styrenesulfonic-*co*-maleic acid) with ester or amide linking groups was synthesized under Steglich conditions. Briefly, 5 g (27.3 mmol of COOH groups) of poly(4-styrenesulfonic-*co*-maleic acid) sodium salt was dissolved in 80 mL of anhydrous dimethyl sulfoxide (DMSO) upon gentle heating. After cooling to room temperature, appropriate amounts of hexadecanol or hexadecylamine (4.9 mmol; 1.19 g or 1.18 g) and coupling agents [DCC, 5.0 mmol, 1.03 g and NHS, 5.0 mmol, 0.58 g (only for hexadecylamine)] and catalytic amounts of DMAP were added. The mixture was stirred at room temperature for 48 h, followed by filtration to remove the byproduct (*N*,*N*′-dicyclohexylurea). The obtained filtrate was dialyzed against distilled water (4 dm^3^—changed four times, 3 days, MWCO 3500), while the product was isolated by freeze-drying.

### Synthesis of Hydrophobically Functionalized PAA

Solid PAA was obtained by freeze-drying its 30% solution in water (purchased from the supplier). Five grams (69.45 mmol of COOH groups) of PAA was dissolved in a minimal amount of anhydrous DMSO (approximately 90–100 mL), followed by the introduction of an appropriate fatty alcohol [27.8 mmol (5.18 g) of dodecanol or 10.4 mmol (2.52 g) of hexadecanol], a coupling agent [DCC, 33.35 mmol (6.87 g) or 12.5 mmol (2.58 g) for dodecanol or hexadecanol, respectively], and a catalytic amount of DMAP. The reaction mixture was stirred at room temperature for 72 h, followed by filtration to remove the byproduct (*N*,*N*′-dicyclohexylurea). The obtained filtrate was dialyzed against distilled water (4 dm^3^—changed five times, 4 days, MWCO 3500), while the product was isolated by freeze-drying.

### Preparation of PLGA Nanoparticles Stabilized by HF-PEs

Briefly, PLGA was dissolved in acetone at a concentration of 10 mg/mL. The obtained organic solution was dropwise added to an appropriate solution of the HF-PE (room-temperature stirring at 850 rpm) to obtain a dispersion of PLGA nanoparticles (final concentration of the PLGA polymer—2 mg/mL). The acetone was removed by overnight stirring at room temperature. Mitomycin-loaded PLGA nanoparticles were prepared using two different approaches with drug molecules dissolved in the organic (acetone) or aqueous phase. The first method consisted of dissolving PLGA and MMC in acetone at concentrations of 10 and 1 mg/mL, respectively. The obtained organic solution was dropwise added to an appropriate solution of the HF-PE (room-temperature stirring at 850 rpm) to obtain a dispersion of PLGA nanoparticles (final concentration of the PLGA polymer—2 mg/mL). The second method constituted the preparation of PLGA solution in acetone (10 mg/mL) and its dropwise addition to water containing appropriate amounts of the HF-PE and mitomycin (0.2 mg/mL). The suspension was stirred (850 rpm) at room temperature. For both approaches, acetone was removed by overnight stirring at room temperature.

### Solubility and Miscibility Parameters of PLGA and Mitomycin

The solubility parameters (δ^(D)^) for the studied drug (MMC) and appropriate polymers (δ^(P)^) were calculated using the Hoftyzer–van Krevelen’s group increment method.^31^ The components of dispersion forces (δ_d_), polar forces (δ_p_), and hydrogen bonding (δ_h_) for the solubility parameters were assessed using both the Hoftyzer–van Krevelen and Hoy approaches. For the Hoy approach, the dispersion forces component (δ_d_) was calculated from the known values of the total solubility parameter (δ_t_) as well as polar forces (δ_p_) and hydrogen bonding (δ_h_) components.
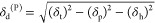
1

The calculated values of the solubility parameters of drugs and polymer matrices were used to calculate the miscibility parameter (Flory–Huggins interaction parameter—χ)
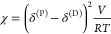
2*V* denotes the molar volume of the studied drug or a single mer (for the polymer), *R* is the universal gas constant, and *T* is the standard temperature in degrees kelvin (298 K). Since the value of the miscibility parameter between two components is lower, the formation of a stable solution or a blend is more likely.

### Characterization of PLGA Nanoparticles

DLS and zeta (ξ) potential studies were performed using a HORIBA SZ-100 nanoparticle analyzer (Retsch Technology GmbH, Germany) at a constant temperature (25 °C) using laser light (λ = 532 nm, 10 mW), and the given values of the solvent viscosity and refractive index (water at 25 °C; 0.8900 mPa·s and 1.3337, respectively) were obtained. The detector scattering angle was chosen automatically during the measurement (at 90° or at 173° for the backscattering mode). To obtain the particle size distribution, the software uses the cumulant method for calculation, and then, the histogram method is applied to obtain the mean and standard deviation of the distribution function from the average of at least five separate measurements from each sample. Electrophoretic mobility was used for ξ potential determination, and each measurement was performed at least five times.

For the determination of the percentage drug loading (DL %) and the encapsulation efficiency (EE %), the absorbance spectra of the encapsulated drug (MMC) were recorded via spectrophotometric measurements in a range of 200 to 600 nm with the use of a UV-1800 (Shimadzu) double beam spectrophotometer with a 1 cm quartz cuvette. The calibration curve (maximum at 354 nm) was recorded in Milli-Q water. Briefly, a suspension of nanoparticles was placed in Eppendorf vials (volume-2 mL) and centrifuged (14,500 rpm, 30 min), followed by 30-fold dilution of the supernatant with distilled water to record UV–vis spectra with the maximal absorbance less than approximately 1. The precipitate was redispersed in a certain volume of distilled water (2 mL), centrifuged, and analyzed to determine the concentration of MMC; no significant signal was found at 354 nm. The well-known DL % and EE % values were calculated using eqs 3 and 4, respectively
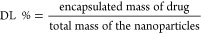
3
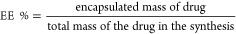
4

For samples 1 and 2, values of EE % and DL % were calculated according to deconvoluted data for a maximum at 359 nm, whereas the main peak at 310 nm was identified as products of mitomycin chemical degradation.

The morphologies and particle sizes of the samples were examined using field emission SEM (Hitachi S-4700 microscope, Tokio, Japan) and TEM (FEI Tecnai G2 20 X-Twin, Hillsboro, Oregon, USA). For SEM analysis, a secondary electron detector and a 10 kV acceleration voltage were applied. Prior to the measurement, suspensions of samples (2 mg/mL PLGA nanoparticles) were placed on the carbon film, followed by drying at room temperature, and covering with gold. Prior to TEM analysis, the samples were diluted 10-fold with distilled water and placed on a copper grid coated with a 3 mm-diameter carbon film (CF200-Cu, electron microscopy sciences, USA), followed by drying at room temperature for 24 h. The transmission electron microscope was operated at an acceleration voltage of 120 kV.^32,33^

### In Vitro Release of MMC from PLGA Nanoparticles

The in vitro drug release experiments were conducted in phosphate-buffered saline (PBS, pH = 7.4, 0.9 w/w % NaCl) at 37 °C using a semipermeable cellulose membrane (Sigma-Aldrich, MWCO 12–14 kDa). Samples were obtained for measurement (UV–vis) at constant periods of time. Free mitomycin was dissolved in PBS prior to placing in dialysis tubing at the desired concentration. The volume of the release medium was kept constant during the measurements. The concentration of MMC in the release medium was determined using calibration curves (Figure S1) in PBS. The obtained release profiles were fitted to different models (Korsmeyer–Peppas, Weibull, and Peppas–Sahlin) according to refs (34) and (35).

## Results and Discussion

### Preparation and Physicochemical Characterization of PLGA Nanoparticles—Influence of the HF-PE Concentration

Newly synthesized hydrophobically functionalized poly(4-styrenesulfonic-*co*-maleic acid) and PAA were utilized for stabilization of the PLGA nanoparticles. The aggregation behavior of the aforementioned compounds was carefully studied in our previous papers.^23,24^ Briefly, hydrophobically functionalized poly(4-styrenesulfonic-*co*-maleic acid) with ester linking groups [e.g., PSS-MA-*g*-C_16_OH (15%)] is more flexible than the analogue derivative with amide bonds [e.g., PSS-MA-*g*-C_16_NH_2_ (15%)] and is prone to self-assembly into aggregates of a diameter of approximately 8 nm at certain concentrations (approximately 22.5 mg/mL: for lower and higher concentrations, only small internal micelle-like objects are observable—see Figure 2 in ref (23)). Hydrophobically functionalized PAA tends to form larger aggregates (hydrodynamic diameter greater than 5 nm) only at extremely high concentrations exceeding 100 mg/mL, and they are completely useless for the fabrication of core–shell nanoparticles using a nanoprecipitation approach. Therefore, we studied the formation of nanoparticles in hydrophobically functionalized poly(4-styrenesulfonic-*co*-maleic acid) derivatives at concentrations ranging from 0.1 to 22.5 mg/mL, while for hydrophobically functionalized PAA, the maximal concentration was set to be 10 mg/mL. According to the literature,^19,36,37^ the final concentration of the PLGA nanoparticle dispersion was tuned to be 2 mg/mL to achieve an appropriate loading content for mitomycin and avoid nanoparticle sedimentation, while the organic phase comprised an acetone solution of the hydrophobic polymer at a concentration equal to 10 mg/mL. It should be noted that the studied colloidal systems were prepared in pure, deionized water to avoid unwanted processes, connected to the influence of ionic strength, especially “salting out” effects on HF-PE macromolecules,^24^ as well as the formation of very large nanoparticles for high salinity of the solution.^36^

**Figure 1:**
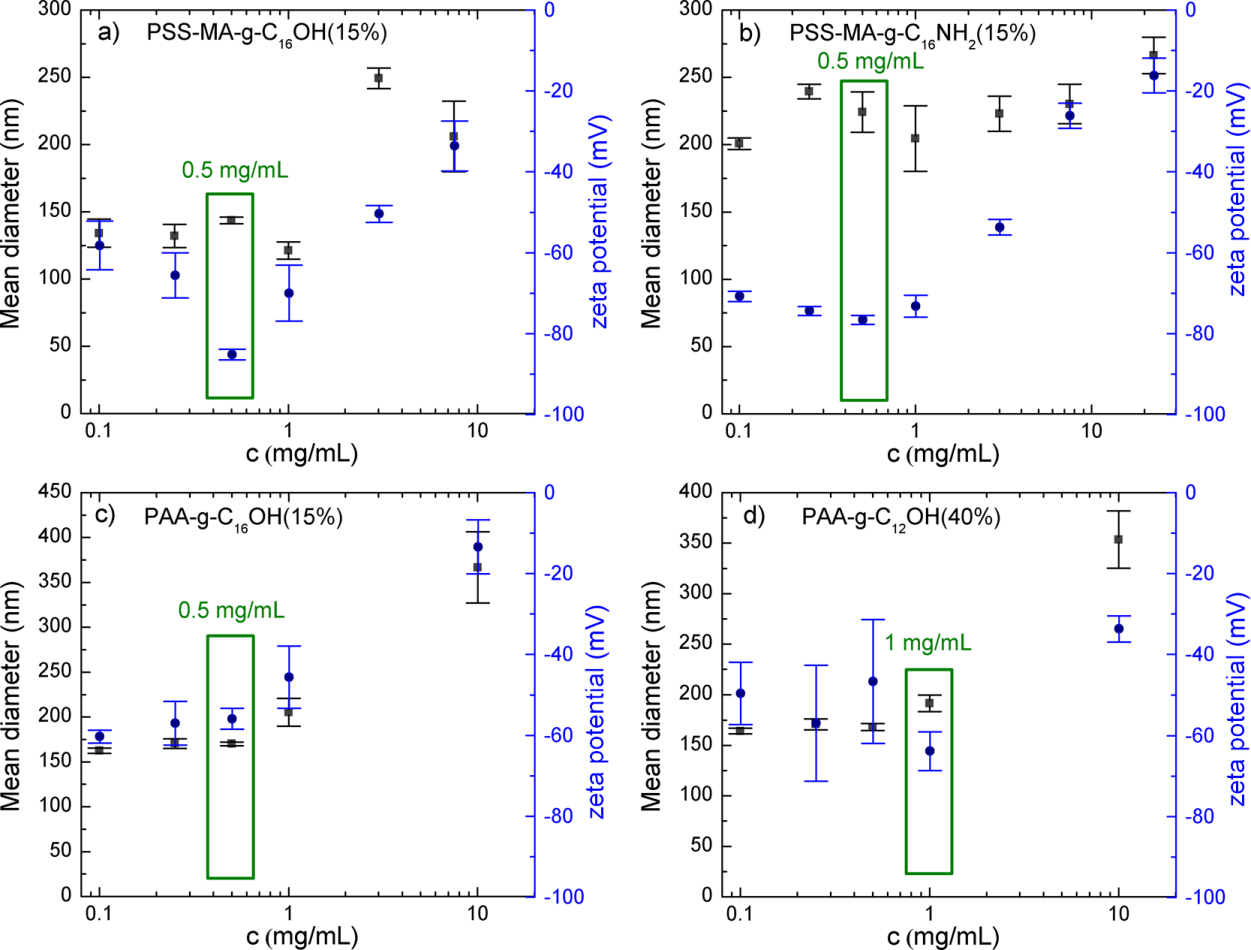
Sizes (black squares) and zeta potentials (blue circles) of PLGA nanoparticles stabilized with HF-PEs: (a) PSS-MA-*g*-C_16_OH (15%), (b) PSS-MA-*g*-C_16_NH_2_ (15%), (c) PAA-*g*-C_16_OH (15%), and (d) PAA-*g*-C_12_OH (40%). Note that the PSS-MA-*g*-C_16_OH (15%) polymer is the most hydrophilic (i.e., the highest HLB value), while the PAA-*g*-C_12_OH (40%) polymer is the most hydrophilic (i.e., the lowest HLB value). Green rectangles mark the maximal optimal concentrations for each HF-PE—minimal value of zeta potentials and stabilization of size.

The optimization studies comprised finding the most appropriate concentration of each HF-PE [PSS-MA-*g*-C_16_OH (15%), PSS-MA-*g*-C_16_NH_2_ (15%), PAA-*g*-C_16_OH (15%), or PAA-*g*-C_12_OH (40%)] for superior nanoparticle characterization: a minimal value of the zeta potential (for negatively charged particles greater than −30 mV or, preferably, less than −50 mV; responsible for colloidal stability via electrostatic repulsion) and nearly stable hydrodynamic diameters with decreasing concentrations (see Figure 1 and Table S1 in the Supporting Information). For PSS-MA-*g*-C_16_OH (15%), PSS-MA-*g*-C_16_NH_2_ (15%), and PAA-*g*-C_16_OH (15%), the optimal concentration was found to be 0.5 mg/mL, while for PAA-g-C_12_OH (40%), it was 1 mg/mL. It should be noted that pure polyelectrolytes and their hydrophobized derivatives showed zeta potentials of approximately 0 mV due to dynamic changes in their conformation and the formation of a nonstable interface.^39−41^ These phenomena were in good agreement with our previous findings obtained using ^1^H NMR investigations—only internal micelles (hydrodynamic diameter of ca. 1–2 nm) were found to be present for broad concentration ranges, but their dimensions were insufficient for measurable values of the zeta potential and observable electrostatic interactions.^23,24^ Conversely, the aforementioned functionalized polyelectrolytes were found to efficiently adsorb at hydrophobic/hydrophilic interfaces, leading to their stabilization via electrostatic repulsion. The higher value of the optimal concentration for PAA-*g*-C_12_OH (40%) in comparison to that of other HF-PEs is connected to its lowest HLB (see Table 1)—the aforementioned compound is too hydrophobic and thus cannot sufficiently stabilize nanoparticles via electrostatic repulsive forces at low concentrations. In general, samples were found to be stable at very broad concentration ranges, even exceeding the optimal values of HF-PEs. Samples containing PSS-MA-*g*-C_16_OH (15%) at a concentration of 22.5 mg/mL were found to be unstable, in contrast to the system stabilized by PSS-MA-*g*-C_16_NH_2_ (15%). According to our previous studies,^23^ the findings are in good agreement with the aggregation behavior of PSS-MA-*g*-C_16_OH (15%)—macromolecules prefer to form larger inter and intramolecular aggregates instead of adsorption on PLGA—and the water interface and stabilization of nanoparticles. Recalling our previous studies upon HF-PEs,^23,24^ for lower concentrations of PSS-MA-*g*-C_16_OH (15%) and other HF-PEs, only the formation of small so-called internal micelles (diameters of approximately 1–2 nm) was observed, so the potential for interfacial stabilization was maintained.

**Figure 2:**
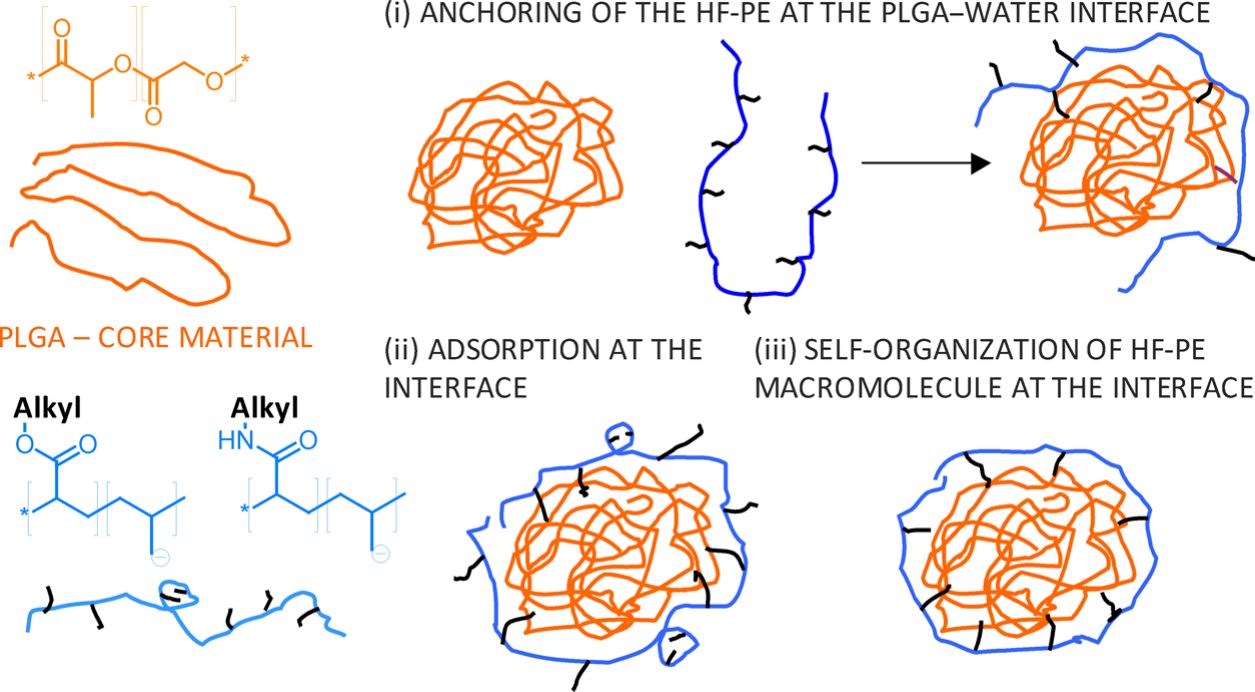
Schematic representation of surface self-organization of HF-PEs.

The mean diameters of the obtained PLGA nanoparticles were strongly dependent on the concentration of HF-PEs: for the highest concentrations (10–22.5 mg/mL), the hydrodynamic diameters varied from approximately 400–450 nm for hydrophobically functionalized PAA to approximately 300 nm for PSS-MA-*g*-C_16_OH (15%) and PSS-MA-*g*-C_16_NH_2_ (15%), while for the lowest and the optimal concentrations, the mean sizes were approximately 150–200 nm [hydrophobically functionalized poly(4-styrenesulfonic-*co*-maleic acid)] or 200–250 nm (derivatives of PAA)—see Figure 1 and Table S1. The aforementioned effect is most likely connected to the kinetics of the process: fast and chaotic adsorption in a concentrated solution of the HF-PE (i.e., concentrations exceeding 1 mg/mL for PAA derivatives and 3 mg/mL for hydrophobically functionalized PSS-MA) results in the formation of large and insufficiently covered polyelectrolyte nanoparticles. It should be emphasized that nonfunctionalized polyelectrolytes or noncharged polymers, such as PAA or poly(vinyl alcohol), are needed at concentrations exceeding around 5 mg/mL in order to obtain PLGA nanoparticles of comparable characteristics (i.e., mean hydrodynamic diameters of ca 100–150 nm).^36^ This effect is especially visible for the most flexible PAA derivatives (ester bonds and only carboxylic acid groups, exhibiting low steric effects)—these findings correspond with relatively low values of zeta potentials for these samples (typically between −10 and −30 mV). In contrast, a lower concentration of the hydrophobized polyelectrolyte (especially around the optimal value) allows for slower and more controlled adsorption on the newly formed interface, leading to better covering with HF-PEs, lower hydrodynamic diameters (150–250 nm), and higher values of zeta potentials [up to approximately −90 mV for PLGA nanoparticles stabilized by PSS-MA-*g*-C_16_OH (15%) at concentrations less than 1 mg/mL)] Similar effects (dependence of the hydrodynamic diameter on the concentration of the stabilizing polymer) were observed for PLGA nanoparticles stabilized by the polyelectrolytes: PAA and poly(styrenesulfonic acid),^19^ as well as noncharged poly(vinyl alcohol).^36^ It should be emphasized that minimal (i.e., optimal) values of zeta potentials reached approximately −85 mV or were even lower only for moderately hydrophobic derivatives [HLB (Davis) ∼ 31, HLB (McGowan) ∼ 12] of PSS-MA. More hydrophobic (HLB ∼ 5–7, regardless of the calculation method) functionalized PAAs allowed for values of minimal zeta potentials of ca. −60 mV to be reached. Moreover, PAA-*g*-C_12_OH (40%)—the most hydrophobic HF-PE (HLB < 6)—showed a minimal value of zeta potentials for its concentration equal to 1 mg/mL in contrast to other studied compounds (the optimal value of zeta potentials for 0.5 mg/mL). The colloidal stability of the obtained systems has been proved using DLS and zeta potential measurements during storage for at least 5 days (see Figures S3 and S4 and Table S1). The aforementioned findings might indicate that the mechanism of the modified polyelectrolyte adsorption comprises a few steps (see Figure 2): (i) anchoring of the HF-PE at the PLGA–water interface; (ii) adsorption of the modified polyelectrolyte at the nanoparticle surface; and (iii) self-organization of the macromolecules to minimize the contact of ionizable groups with hydrophobic fragments.

It seems that the first step is the fastest and depends mostly on the presence of hydrophobic groups in the polyelectrolyte structure, regardless of their amount in the macromolecule. Only a small number of such anchoring points per HF-PE molecule is sufficient for further stability, covering the PLGA–water interface (polyelectrolytes with 15% hydrophobization and higher HLB values were found to form nanoparticles of lower zeta potential values, i.e., more stable ones). The following processes are much slower than anchoring and are generally more efficient for stronger electrolyte groups (e.g., sulfonate) than for weaker electrolyte groups (e.g., carboxylate).

Numerous macromolecules or low-molecular-weight amphiphilic compounds could be used for the stabilization of hydrophobic polymer-borne colloidal nanoparticles. In general, the aforementioned colloidal systems comprise core–shell structures with hydrophobic, solid cores and stabilizing shell layers of hydrophilic or (better) amphiphilic characteristics. For drug delivery systems, biocompatible and biodegradable polymers are used as hydrophobic matrices, such as different polyesters (PLA, PLGA, and PCL), some polysaccharides (chitosan), and proteins [poly(glutamic acid)]. In contrast, typical stabilizing agents, such as surfactants [e.g., bis(2-ethylhexyl) sodium sulfosuccinate], nonionizable polymers [e.g., poly(vinyl alcohol)], or polyelectrolytes [e.g., PAA or poly(styrenesulfonic acid)], lead to the formation of unstable colloidal systems.^19^ Moreover, some of them have not yet been fully tested for biomedical applications (especially synthetic polyelectrolytes) and must be used at high concentrations (e.g., greater than 2 mg/mL) to provide sufficient colloidal stability, especially when relatively low-molecular-weight compounds are used.^36,37^ The mechanism of the aforementioned core–shell nanostructure formation comprises adsorption of the stabilizing agent (especially the water-soluble polymer) at the hydrophobic polymer/aqueous solution interface. This process is reversible, so desorption of the solubilizing agent can also occur, leading to unwanted processes, such as agglomeration, sedimentation, and finally destabilization of the colloidal system. Considering these drawbacks, a unique class of HF-PEs were carefully studied as stabilizing materials for PLGA nanoparticles as mitomycin carriers. The aforementioned compounds comprise both ionizable groups, for example, carboxylic acid or styrenesulfonate moieties, and shorter or longer hydrophobic side chains.^38^ Charged groups are responsible for stabilization by repulsive electrostatic forces, while hydrophobic side chains can anchor to the surfaces of hydrophobic nanoparticles and provide additional attractive interactions between polyelectrolytes and core-forming materials. Conversely, these side chains can also sterically stabilize the charged particles, further increasing the stability of the particles.

### PLGA and Mitomycin Solubility and Miscibility Studies

The compatibility between the drug molecule and the polymeric matrix could be assessed by means of solubility (δ) and Flory–Huggins interaction (miscibility) parameters (χ).^14^ Although the calculation of solubility and miscibility parameters for polymers considers only a single building unit (mer), there exists a nondirect correlation between the molecular weight and the capacity of the host polymeric matrix. Generally, with the increasing polymer molecular weight, the solubility of drug molecules in the polymeric matrix also increases, especially for moderate values of the Flory–Huggins interaction parameter. This effect is possibly connected to the growing probability of forming different subdomains with the increasing polymer chain length.^46^ These parameters have been found to play an important role in the prediction of the aqueous and micellar solubility of the drug,^47^ release profiles,^48,49^ solubilization locus within nanocarriers,^18,46^ and thermodynamic properties of drug–polymeric matrix systems.^49,50^ Compatibility and solubility parameter studies might enable us to distinguish two possibilities of interaction between the drug and the host polymeric matrix: molecular dissolution of the payload in the polymer for systems with high compatibility or accumulation of the drug molecules at the external or internal interfaces (e.g., borders between crystalline and amorphous subdomains or the highly developed porous external layer of the polymer) when the compatibility degree is insufficient.^18^ Generally, for systems with equal degrees of hydrogen bonding, an ideal miscibility is assumed for χ < 0.5, but complete immiscibility occurs when the absolute difference between the drug and the polymer exceeds 10 MPa^0.5^.^31^

The aforementioned requirements are roughly fulfilled for MMC in the PLGA matrix: the difference in the hydrogen bonding increment is smaller than 3 MPa^0.5^ for both Hoy’s and Hoftyzer–van Krevelen’s models. According to the calculated values of solubility and miscibility parameters (see Table 2), MMC is compatible with PLGA—χ values are lower than 0.5 (all the studied approaches: Hoy, Hoftyzer–van Krevelen, and cohesive energy by Fedors). These results indicate that mitomycin molecules are solubilized within nanoparticles cores. Similar observations were obtained for hexadecafluoro zinc(II) phthalocyanine in methoxypoly(ethylene oxide)-*b*-poly(l-lactide) micelles [the solubilization locus is outside the core despite high hydrophobicity of the payload due to high incompatibility with poly(l-lactide) chains]^15^ as well as tetra *tert*-butyl zinc(II) phthalocyanine in methoxypoly(ethylene oxide)-*b*-poly(d,l-lactide) and in methoxypoly(ethylene oxide)-*b*-PCL micelles^18^ (in particular, accumulation of phthalocyanine molecules at the boundary between the two subdomains of different flexibilities, situated within the hydrophobic micelle core). Numerous drug delivery systems, including polymeric micelles and nanoparticles, layer-by-layer nanocapsules, and other core–shell structures, consist of hydrophilic external layers to prevent opsonization.^1,2^ Such an approach very often includes the use of amphiphilic blocks or grafted copolymers, with hydrophilic fragments comprising appropriate water miscible fragments, for example, poly(ethylene oxide), and hydrophobic or amphiphilic blocks, enabling anchoring to the carrier. The hydrophobic fragment of the amphiphilic copolymer should be compatible with the polymeric material, constituting a host space for drug molecules.^46^ Conversely, core–shell-type nanoparticles should possess an internal polymeric structure of very good compatibility with the drug to provide optimal characteristics of the aforementioned nanocarrier (controlled release profiles, appropriate colloidal and chemical stability, and low susceptibility to unwanted processes during systemic circulation).^1^ Based on the above, our system—core–shell nanoparticles with an internal PLGA matrix as the host material for MMC stabilized by HF-PEs—fulfills the aforementioned requirements: the core material is highly compatible with the drug, while the shell-forming polymer is charged to provide stabilization.

**Table 2:** Solubility (δ) and Miscibility (χ) Parameters of PLGA and Mitomycin

mitomycin			PLGA			
δ_d_	δ_p_	δ_h_	δ_d_	δ_p_	δ_h_	
MPa^0.5^	MPa^0.5^	MPa^0.5^	MPa^0.5^	MPa^0.5^	MPa^0.5^	χ
Hoy’s Method						
13.0	15.0	12.7	14.1	13.7	10.0	0.24
Hoftyzer–van Krevelen’s Method						
21.7	7.4	13.9	18.1	10.9	12.5	0.43
Cohesive Energy Method by Fedors						
22.6			23.6			0.02

Solubility parameters (δ) could be utilized in an increasing number of scientific and engineering fields for estimating interaction capacities and solubilities between various organic liquids, polymers, and even some solids.^42−45^ The solubility parameter and its increments describing dispersion forces (δ_d_), polar forces (δ_p_), and hydrogen bonding (δ_h_) were introduced to predict the solubility of different polymers in organic solvents, as well as the possibility of polymer blend formation.^31^ The most important advantages of solubility parameter usage comprise their universal character (applicable for liquid, gaseous, and solid organic substances), the possibility of their determination utilizing both experimental (analysis of thermodynamic properties and molecular modeling) and theoretical (group increment methods) approaches as well as their simple combination with other physicochemical parameters (cohesive energy, heat of vaporization, etc.). Recently, solubility parameters have been used to predict the formation of cocrystals^45^ and amorphous solids,^43,44^ interactions between active pharmaceutical ingredients and carrier materials,^46^ and the internal structures of polymeric micelle cores loaded with zinc(II) phthalocyanine derivatives.^18^

### Mitomycin-Loaded PLGA Nanoparticles—Synthesis and Characterization

An ideal nanocarrier should be colloidally stable, provide chemical stability of the payload, and control its release upon arrival at the place of action or during systemic circulation. In general, the first feature is directly or indirectly measured using various techniques (e.g., DLS, atomic force microscopy, SEM, and TEM) and described by parameters, such as hydrodynamic diameter, dry-state size, polydispersity, or zeta potential.

**Figure 3:**
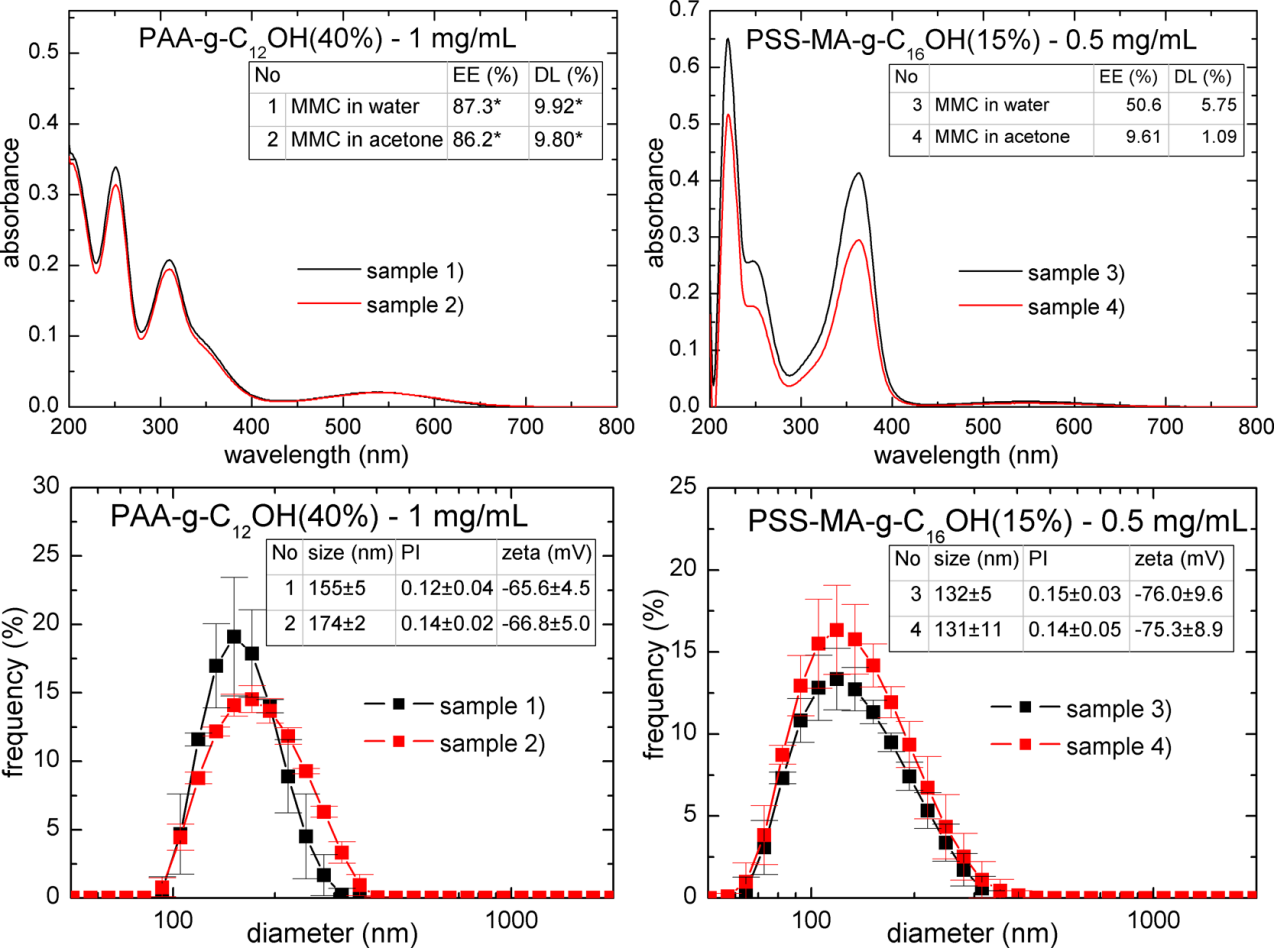
UV–vis spectra (top) and DLS size distribution graphs (bottom) of MMC-loaded PLGA nanoparticles; EE (%)—encapsulation efficiency and DL (%)—drug loading content.

Our investigations included the preparation of PLGA nanoparticles stabilized by PAA-C_12_OH (40%) at a concentration of 1 mg/mL and PSS-MA-C_16_OH (15%) at a concentration of 0.5 mg/mL by dissolving MMC in both water or acetone (systems 1 and 2, as well as systems 3 and 4, respectively, in Figure 3). Recalling the preparation methodology (see the Experimental section) systems 2 and 4 were prepared by dissolving both MMC and PLGA in acetone, followed by dropwise addition into water containing the HF-PE. Systems 1 and 3 were prepared in the following manner: MMC and HF-PE were dissolved in water, followed by the introduction of PLGA solution in acetone. All of the studied systems were characterized by mean diameters of approximately 150–175 nm (systems 1 and 2) and approximately 130 nm (systems 3 and 4), as well as a reasonably low polydispersity (less than 0.3)—see Figure 3. Loading of PLGA nanoparticles with MMC has a negligible effect on their zeta potential values [ca −60 mV and ca. −80 mV for PAA-C_12_OH (40%) and PSS-MA-C_16_OH (15%) as shell-forming materials, respectively], indicating that the drug is loaded within the core rather than adsorbed within the shell layer. These findings are in good agreement with the excellent compatibility between the core-forming material (PLGA polymer) and the drug (MMC)—values of the χ parameter are less than 0.5. It should be noted that for both HF-PEs [PAA-C_12_OH (40%) and PSS-MA-C_16_OH (15%)], higher values of the encapsulation efficiency (EE %) and drug loading content (DL %) were observed for samples prepared by dropwise addition of MMC and PLGA solution in acetone (see Figures 3 and S2 for calibration curves in water). Recalling, values of EE % and DL % for samples 1 and 2 were calculated according to deconvoluted data for a maximum at 359 nm [the aforementioned numbers are noted with an asterisk (*) in Figure 3]. This effect is most likely connected to the kinetics of nanoprecipitation–MMC has a greater tendency to be entrapped within the PLGA core microenvironment when both compounds [MMC and PLGA] are dissolved in the same solvent. It should be noted that only PSS-MA-C16OH (15%) exhibited chemical stability of the encapsulated and released MMC–PLGA nanoparticles, stabilized by PAA-C12OH (40%), leading to fast degradation of the drug (presence of the maximum at approximately 310 nm in the UV–vis spectrum, attributed to degradation products such as *cis-* and *trans*-isomers of 2,7-diamino-1-hydroxymitosene). PSS-MA-C_16_OH (15%) consists of both strong (styrenesulfonate sodium salt) and weak (partially neutralized carboxylic acid) electrolyte groups, leading to a “buffering” effect and reducing the possibility of strongly acidic group formation. Conversely, PAA-C_12_OH (40%) possesses only carboxylic acid groups, so the interfacial acidity is large and could constitute catalysts for acidic hydrolysis of MMC.

In order to compare our results with studies carried out by other researchers, it is needed to emphasize that MMC is a chemotherapeutic agent that is considered to be amphiphilic/hydrophilic (logP value = −0.4—see Table 1) and can be combined with other drugs (e.g., doxorubicin).^20−22^ MMC has been successfully loaded or coloaded into polymer lipid nanoparticles,^20^ obtained via hot oil phase emulsification (mitomycin dispersed in the oil phase), polymer–lipid hybrid nanoparticles (thin film method, followed by dialysis from a dimethylformamide (DMF)–water mixture, with mitomycin initially dissolved in tetrahydrofuran),^51^ micelles of stearic acid-grafted chitosan oligosaccharide modified with polyethylene glycol (PEG) chains (mitomycin dissolved in the aqueous phase),^52^ and micelles of deoxycholic acid chitosan-grafted poly(ethylene glycol) methyl ether (emulsification method, with mitomycin dissolved in acetone).^53^ Due to the hydrophilic/amphiphilic characteristics of MMC, the preparation of nanocarriers could comprise its dissolution in both organic (e.g., acetone, tetrahydrofuran, or DMF) and aqueous phases. The latter approach is particularly useful when building blocks of nanocarriers possess an amphiphilic character (e.g., comprise both hydrophilic/ionizable groups and hydrophobic blocks/fragments).^52^ One of the main drawbacks of MMC is its high susceptibility to chemical degradation upon exposure to chemical (e.g., acidic or basic conditions)^54^ or enzymatic (e.g., presence of NADPH–cytochrome P-450 reductase or xanthine reductase^55^) factors. The main degradation products consist of *cis*- and *trans*-isomers of 2,7-diamino-1-hydroxymitosene, exhibiting an absorbance maximum of approximately 310 nm (pure MMC—364 nm). This is why our studies comprised novel core–shell PLGA nanoparticles—carrier systems that have not been used for MMC encapsulation—characterized by excellent drug–core material compatibility and superior tenability of properties.

**Figure 4:**
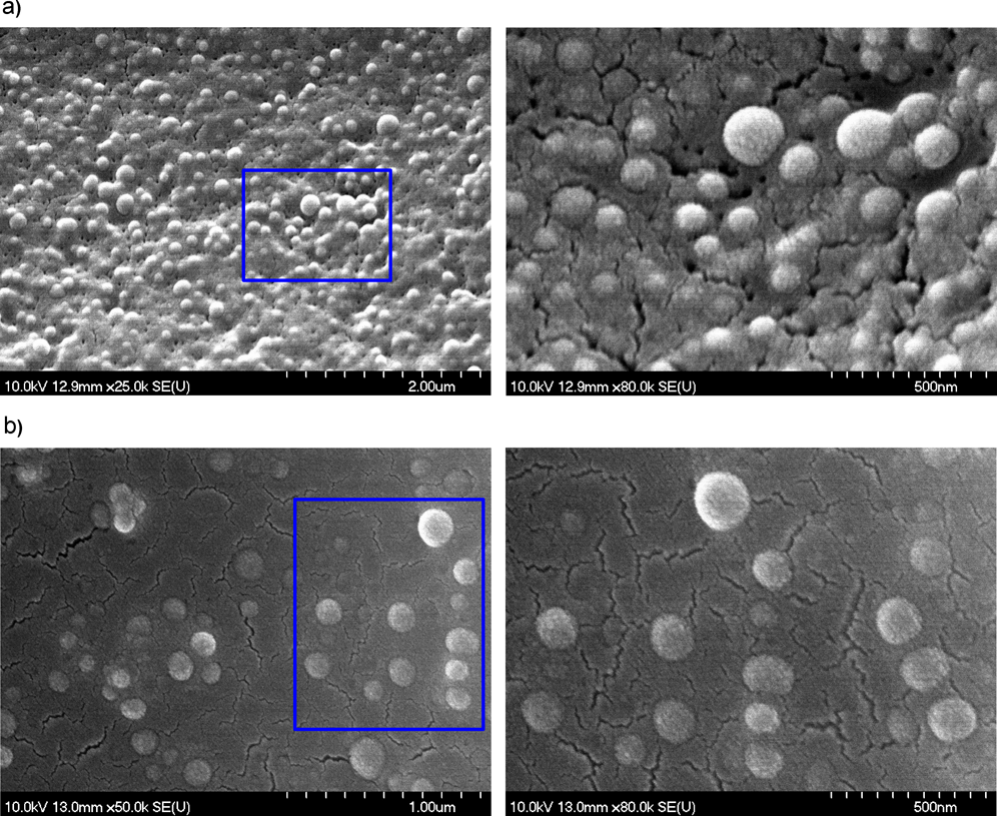
SEM images of MMC-loaded PLGA nanoparticles stabilized by (a) PSS-MA-g-C_16_OH (15%) (system 4 according to Figure 3) and (b) PAA-C_12_OH (40%) (system 2 according to Figure 3). The magnified fragments at different angles are marked with green rectangles.

**Figure 5:**
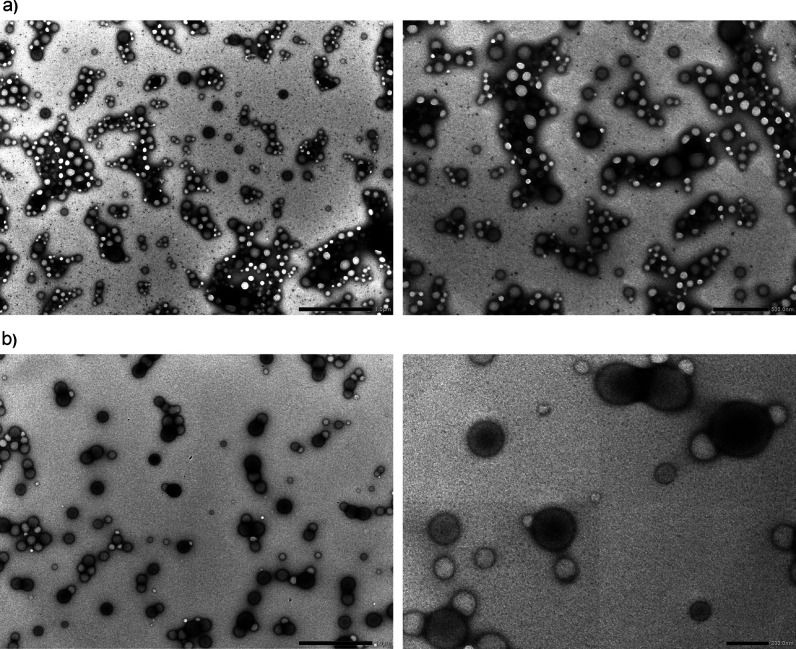
TEM images of MMC-loaded PLGA nanoparticles stabilized by (a) PSS-MA-g-C_16_OH (15%) [system 4 according to Figure 3, scale bars: 1 μm (left) and 500 nm (right)] and (b) PAA-C_12_OH (40%) [system 2 according to Figure 3, scale bars: 500 nm (left) and 200 nm (right)]. The scale bars were tuned for optimal resolution of the nanoparticles.

The morphology of the obtained MMC-loaded nanocarriers was assessed using SEM and TEM (see Figures 4, 5, and S5). See also Figure S6 for macroscopic images. Electron microscopy images confirmed the DLS results—the obtained nanocarriers were spherical-shaped with only a slightly rough surface. The mean diameters of these well-defined spherical objects were nearly uniform, corresponding to low values of polydispersity indices (PD < 0.3). For TEM images, the mean size distribution of circular objects was reasonably larger than that obtained using SEM, possibly due to sample dilution prior to the analysis, although it also met the requirements for low polydispersity. The circular objects in TEM images showed differences between electron beam absorptivity between their center and the outer layer, which could correspond to the core–shell structure of PLGA nanoparticles. Similar observations were obtained for nanoparticulate poly(4-styrenesulfonic-*co*-maleic acid) loaded with hydrophobic drugs.^32^ The thickness of the postulated shell layer is very low (ca 1–2 nm), indicating good agreement with the HP-PE behavior (formation of only small, stable “internal micelles” of diameter less than 2 nm, which might be present on the surfaces of nanoparticles).^23,24^ Moreover, no internal crystallites are visible on the TEM images, indicating that MMC is molecularly dissolved in the polymeric matrix, corresponding with the very good compatibility between MMC and PLGA. It should be noted that the difference in mean hydrodynamic diameters between particular samples [e.g., stabilized by PSS-MA-g-C_16_OH (15%) and PSS-MA-g-C_16_NH_2_ (15%)] is also visible in both SEM and TEM images [PLGA nanoparticles, stabilized by PSS-MA-g-C_16_NH_2_ (15%), are significantly larger than those with PSS-MA-g-C_16_OH (15%) shell layers—see Figures 3–5 and S5, as well as Table S1 for data], despite the difference in concentrations of the solution dipped on the surface (2 mg/mL for SEM or 0.2 mg/mL for TEM) and in the preparation methods (covering with a thin gold layer prior to SEM measurements). The obtained results (DLS, SEM, and TEM) showed the high potential of the optimized preparation methods for PLGA nanoparticles as MMC delivery systems—their shape and size uniformity, stability, and property tunability using appropriate HF-PEs at certain concentrations.

### In Vitro Release Studies—Influence of the Polyelectrolyte Shell Layer

The release kinetics of the bioactive compound under certain conditions are amenable for determining whether the nanocarriers or microcarriers are suitable for the chosen method of administration. In general, it should be emphasized that the release profiles are dependent on numerous factors connected to the payload, the formulation, and the release medium. The in vitro release characteristics might allow us to gain crucial information about the physicochemical characteristics of the carrier system and the payload, including chemical degradation, erosion, diffusion, and responsivity to external stimuli.^2^

**Figure 6:**
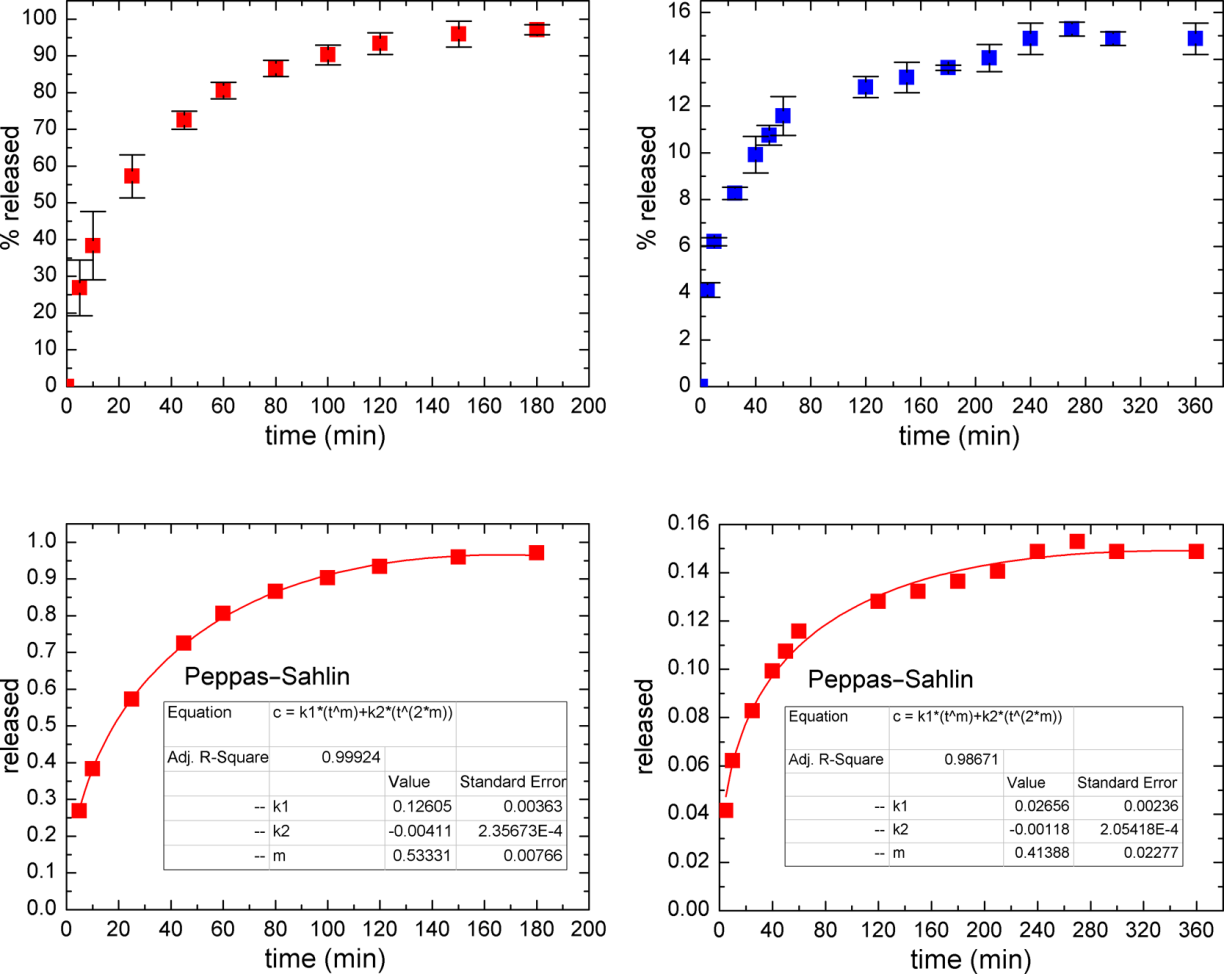
In vitro release profiles (up) of MMC in PBS at 37 °C: pure MMC (left panel) and MMC loaded into PLGA nanoparticles (right panel, system 3 according to Figure 3). Down: data fitted to the best Peppas–Sahlin model.

To describe the mechanism of drug release from the carrier, it is possible to fit the data points (the amount of the released drug against release time) to well-known models, such as Korsmeyer–Peppas, Weibull, and Peppas–Sahlin. The Korsmeyer–Peppas model is a semiempirical power law equation, considering the influence of the shape of the polymer matrix (such as film, cylinder, or sphere) on the release profile.^35^ According to this model, k_m_ is the kinetic constant and n is the diffusion dissolution index [for spherical particles, *n* = 0.42 (diffusion-controlled mechanism); *n* = 1 for the case II relaxation-controlled mechanism; and 0.42 < *n* < 1 for the combined mechanism)]. The Weibull equation is a general empirical formula, where *a* is the time scale of the process and *b* is the shape parameter (shape of the release curve is exponential if *b* = 1, parabola if *b* < 1, or sigmoid if *b* > 1). The kinetic formula reported by Peppas–Sahlin combines all of the possibilities of the Korsmeyer–Peppas model and specifies the diffusion and relaxation contributions to the drug dissolution process, where *k*_1_ is the Fick diffusion contribution, *k*_2_ is the case II relaxation contribution, and *m* is the diffusion exponent (spherical shaped: *m* = 0.43, Fick diffusion mechanism; *m* = 0.85, case II relaxation transport mechanism; and 0.43 < *m* < 0.85, anomalous transport mechanism).^34,35^ To compare different models with each other, the parameter called “Adjusted R^2^” was used as an indicator of the fitting quality to avoid misinterpretation of equations with varying numbers of constants (see Figures 6 and S7 and Table 3). The best fitting was obtained for the Peppas–Sahlin model (the value of adjusted R^2^ was closest to 1) for both free mitomycin and system 3. According to this equation, the loaded system was in good agreement with the sphere-shaped model (*m* value close to 0.43). Considering the amphiphilic character of MMC, the Peppas–Sahlin model appeared to also be the best in fitting the release of the free drug, although the difference in adjusted R^2^ parameters between the Peppas–Sahlin and Weibull equations was negligible. It should be emphasized that only approximately 15% of the encapsulated drug underwent fast (ca 6 h) release. The remaining 85% of MMC underwent gradual release, connected to the gradual degradation of the polymer matrix, as well as the difficult diffusion of mitomycin from the core of the carrier. In contrast, the linear fragment (up to approximately 10% of the released drug) might originate from the MMC molecules adsorbed at the nanocarrier surfaces.^35^

**Table 3:** Parameters of MMC Release Determined by Fitting Several Kinetic Equations (MMC-Loaded Nanoparticles – System 3 According to Figure 3)

	Korsmeyer–Peppas			Weibull			Peppas–Sahlin			
	k_m_	n	Adj R^2^	a	b	Adj R^2^	k_1_	k_2_	m	Adj R^2^
system	min^-n^						min^-m^	min^–2m^		
free MMC	0.2082	0.3113	0.9527	10.17	0.6845	0.9985	0.1260	–4.11*10^–3^	0.5333	0.9992
MMC in nanoparticles	0.0388	0.2414	0.9515	25.95	0.2569	0.9544	0.0266	–1.18*10^–3^	0.4139	0.9867

Solubility and miscibility parameters significantly affect the release profiles of the drug from the polymeric carrier matrix in an appropriate medium. MMC itself is a moderately water-soluble compound, so it needs ca 3 h to completely diffuse through the membrane. With the increase in the compatibility degree between the polymer and the payload, the release rates decrease, although the influences of the drug-to-polymer ratio and polymer mean molecular weight are also observed.^49^ Our system—MMC loaded into PLGA—should release drug molecules at slow or moderate rates due to the miscibility parameter (χ less than 0.5), showing complete compatibility and relatively similar hydrogen bonding increments (difference is smaller than 3 MPa^0.5^). Moreover, higher release rates should be observed for polymers with higher molecular weights due to their more complicated internal structures. It is also postulated (due to the presence of no crystalline forms of MMC in the TEM images) that the release process is controlled by the diffusion of the drug from the polymer matrix, rather than dissolution of crystallites.^32^ It should be emphasized that PLGA nanoparticles are proven for their excellent sustained release properties. For example, PLGA–PEG nanoparticles, loaded with dexamethasone, allowed sustained release within ca 12 days, therefore reducing side effects, connected with large single doses of the drug.^56^

## Conclusions

Our investigations showed the high potential of PLGA nanoparticles stabilized by HF-PEs as delivery systems for MMC. The first step consisted of optimization studies for different PAA [PAA-C_12_OH (40%) and PAA-C_16_OH (15%)] and poly(4-styrenesulfonic-*co*-maleic acid) [PSS-MA-*g*-C_16_NH_2_ (15%) and PSS-MA-g-C_16_OH (15%)] derivatives—these experiments allowed us to clearly show that sufficient hydrophilicity (i.e., HLB value greater than 6) is needed to sufficiently stabilize PLGA nanoparticles at minimal concentrations (0.5 mg/mL), regardless of the type of the HF-PE. It is also possible to use less hydrophilic hydrophobically functionalized PEs (i.e., HLB value less than 6), but the minimal optimized concentration was higher (1 mg/mL). Moreover, PLGA nanoparticles stabilized with more hydrophilic HF-PEs (HLB value greater than 7) exhibited significantly lower minimal values of zeta potentials (approximately—85 mV) compared to those with a modified PE of higher hydrophobicity (ca −60 mV). Our studies showed that further reduction of HF-PEs to less than the optimal value is possible but could result in slightly less stable systems. It should be emphasized that the flexibility of the chemical bonds between the backbone and side chains has a significant impact on the PLGA nanoparticle hydrodynamic diameters: nanocarriers stabilized by PSS-MA-g-C_16_OH (15%) with flexible ester bonds are characterized by significantly smaller hydrodynamic diameters compared to those stabilized by PSS-MA-g-C_16_NH_2_ (15%) with more rigid amide bonds. Our findings of DLS measurements were also confirmed using TEM and SEM techniques, showing spherical (SEM) or circular (TEM) objects of the appropriate size, polydispersity, and morphology (core–shell nanoparticles). PLGA was chosen as a host material for amphiphilic mitomycin due to its moderately water-soluble character and excellent compatibility with the drug molecules [value of the miscibility parameter χ is less than 0.5 for only a slight difference (less than 3 MPa^0.5^) between appropriate hydrogen bonding increments (δ_h_) of the solubility parameter]. The aforementioned findings were confirmed by the negligible effect of MMC loading into PLGA nanoparticles on the zeta potential values, indicating that the drug is incorporated inside the core rather than adsorbed within the shell layer. MMC was found to be chemically unstable in colloidal suspensions of PAA derivative-stabilized PLGA nanoparticles—most likely due to high hydrolysis rates connected with weak electrolyte carboxylic acid groups; the UV–vis spectra showed the presence of *cis*- and *trans*-isomers of 2,7-diamino-1-hydroxymitosene (common degradation products of MMC) in both supernatants after nanoparticle preparation, as well as the release medium. Only PLGA nanoparticles stabilized by hydrophobically functionalized poly(4-styrenesulfonic-*co*-maleic acid) protected MMC against preliminary leakage, chemical degradation, or colloidal destabilization. The release profiles of free and encapsulated MMC exhibited the best fitting for Peppas–Sahlin, showing good agreement with the found shape of the nanocarriers (spherical), the amphipilic character of the drug, and the solubility/miscibility parameters studies (good compatibility between the polymeric matrix and the payload). The present contribution could open a new possibility of selecting more efficient building and stabilizing blocks for functional polymeric dispersions toward their biomedical application as carrier systems for amphiphilic and chemically instable drugs, showing crucial features for consideration in their future research.

## Supporting Information Available

Additional results including data of nanoparticle size and its distribution using DLS; nanocarrier colloidal stability using DLS; SEM images of PLGA nanoparticles without mitomycin; and calibration curves for mitomycin in water and PBS buffer (PDF)
