# Supplementary material for: Rational Mitomycin Nanocarriers Based on Hydrophobically Functionalized Polyelectrolytes and Poly(lactide-co-glycolide)
Source: Langmuir. 2022 Apr 20;38(18):5404–17. doi: 10.1021/acs.langmuir.1c03360 (PMC9097536; doi:10.1021/acs.langmuir.1c03360)
Supplement: Supplementary file 1 — la1c03360_si_001.pdf [file la1c03360_si_001.pdf]

Supporting Information

Rational Mitomycin Nanocarriers Based on Hydrophobically Functionalized Polyelectrolytes and Poly(Lactide-co-Glycolide)

Łukasz Lamch [a)] *, Kazimiera A. Wilk [a)], Imre Dékány [b)], Ágota Deák [b)], Viktória Hornok [b)], László Janovák [b)]

[a)] Department of Engineering and Technology of Chemical Processes, Faculty of Chemistry, Wrocław University of Science and Technology, Wybrzeże Wyspiańskiego 27, 50-370 Wrocław, Poland; kazimiera.wilk@pwr.edu.pl

[b)] University of Szeged, Department of Physical Chemistry and Materials Science, H-6720, Rerrich Béla tér 1, Szeged, Hungary; i.dekany@chem.u-szeged.hu, agotadeak@chem.u-szeged.hu, vhornok@chem.u-szeged.hu, janovakl@chem.u-szeged.hu

* To whom correspondence should be addressed

phone: +48 71 3203447, fax: +48 71 3203678

email address: lukasz.lamch@pwr.edu.pl,

Table of contents Page

1. Calibration curves for mitomycin in water and PBS buffer S2

2. Representative graphs for micelles size and its distribution by DLS S4

3. Polymeric micelles colloidal stability by DLS S5

4. SEM images of PLGA nanoparticles without mitomycin S7

5. Macroscopic images S8

6. Fitting release data to Korsmeyer-Peppas and Weibull models S9

1. Calibration curves for mitomycin in water and PBS buffer

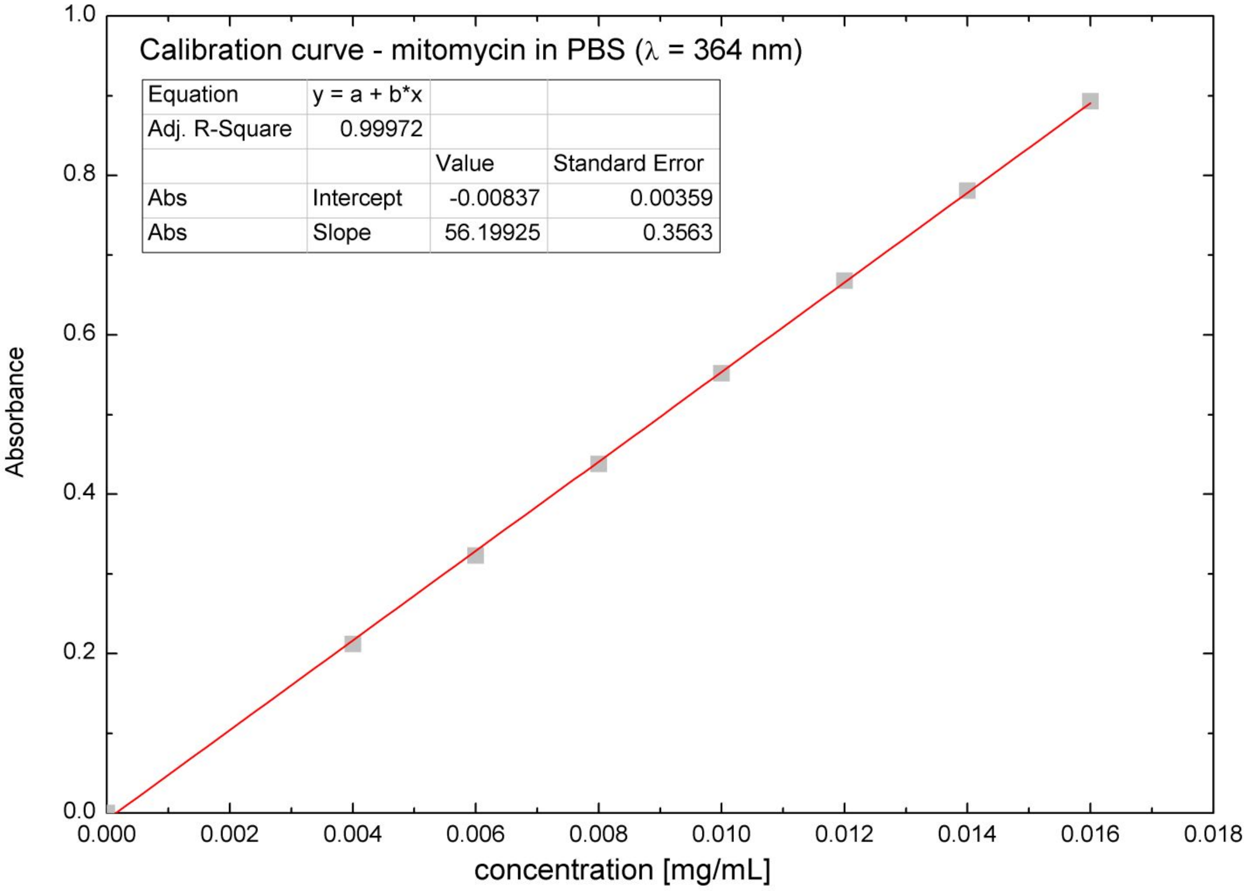

Figure S1. Calibration curve for mitomycin in PBS buffer (pH = 7.4).

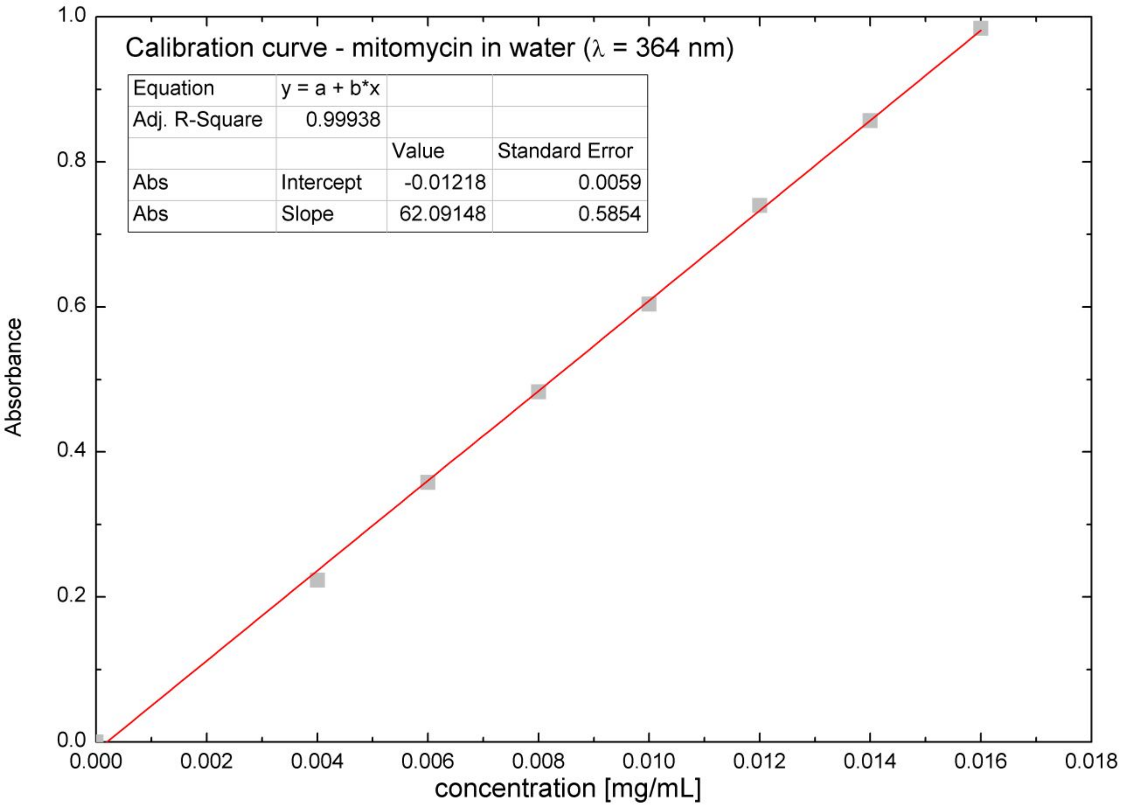

Figure S2. Calibration curve for mitomycin in water.

2. Representative graphs for micelles size and its distribution by DLS

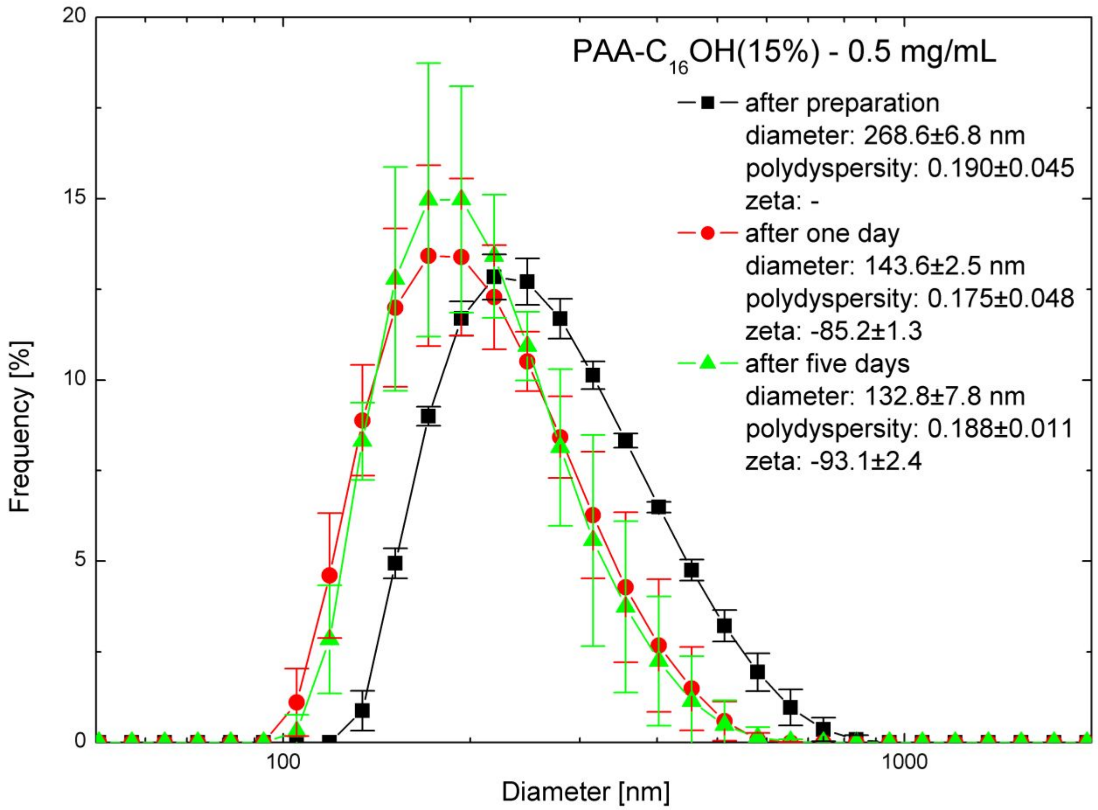

Figure S3. Size and its distribution by DLS for PAA-C_{16}OH(15%) stabilized PLGA nanoparticles (without mitomycin).

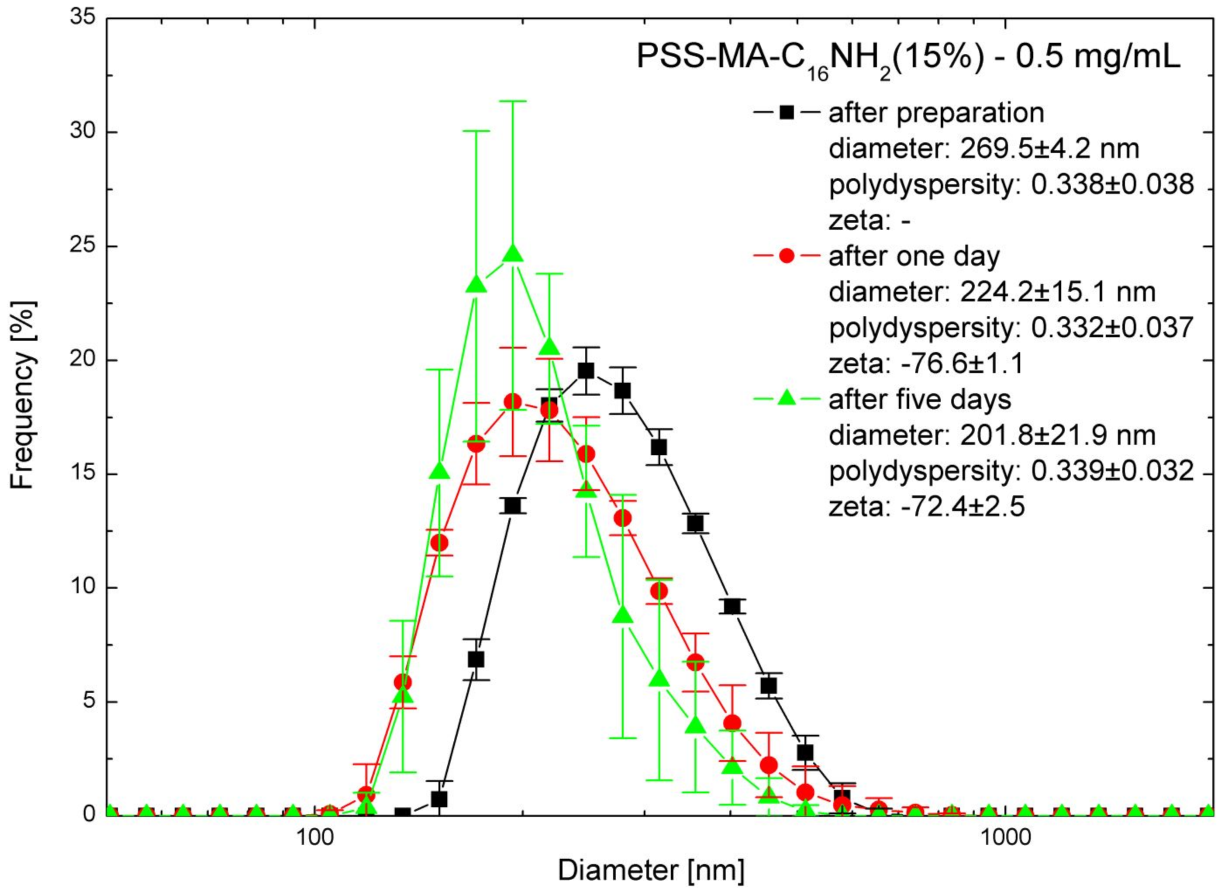

Figure S4. Size and its distribution by DLS for PSS-MA-$C_{16}NH_2$(15%) stabilized PLGA nanoparticles (without mitomycin).

3. Polymeric micelles colloidal stability by DLS

Table S1**.** Colloidal stability of the studied nanoparticles without mitomycin.

**Polymer**	**C [mg/mL]**	**After preparation**			**After one day (evaporation of acetone)**		
		**size [nm]**	**PI**	**zeta**	**size [nm]**	**PI**	**zeta**
PAA-C_{12}OH(40%)	1	232.0±7.7	0.196±0.106	-	191.3±8.2	0.172±0.061	-63.9±4.8
PAA-C_{12}OH(40%)	10	428.6±23.2	0.246±0.038	-28.0±3.5	353.5±28.3	0.249±0.063	-33.7±3.2
PAA-C_{16}OH(15%)	1	268.6±6.8	0.190±0.045	-	205.1±15.5	0.239±0.034	-45.6±7.7
PAA-C_{16}OH(15%)	10	431.7±31.5	0.336±0.042	-19.0±2.9	366.7±39.6	0.670±0.277	-13.4±6.7
PSS-MA-$C_{16}NH_2$(15%)	0.5	269.5±4.2	0.338±0.038	-	224.2±15.1	0.332±0.037	-76.6±1.1

PSS-MA-$C_{16}NH_2$(15%)	1	258.0±20.9	0.268±0.070	-56.6±3.71	204.5±24.4	0.274±0.042	-73.2±2.7
PSS-MA-$C_{16}NH_2$(15%)	3	286.1±25.9	0.303±0.031	-46.8±5.1	223.0±13.0	0.270±0.054	-53.7±1.9
PSS-MA-$C_{16}NH_2$(15%)	7.5	288.1±5.8	0.242±0.065	-29.3±4.8	230.2±14.7	0.201±0.078	-26.1±3.1
PSS-MA-$C_{16}NH_2$(15%)	22.5	320.0±21.3	0.268±0.059	-12.7±2.8	266.3±13.6	0.279±0.055	-16.2±4.3
PSS-MA-$C_{16}OH$(15%)	0.5	184.3±8.0	0.128±0.035	-	143.6±2.5	0.175±0.048	-85.2±1.3
PSS-MA-$C_{16}OH$(15%)	1	164.8±2.9	0.113±0.065	-53.1±11.3	121.2±6.5	0.143±0.036	-70.0±6.9
PSS-MA-$C_{16}OH$(15%)	3	185.6±5.1	0.105±0.054	-51.6±2.4	249.2±7.6	0.159±0.073	-50.4±2.1
PSS-MA-$C_{16}OH$(15%)	7.5	256.3±14.6	0.230±0.038	-38.6±2.7	206.0±26.3	0.171±0.106	-33.6±6.2
PSS-MA-$C_{16}OH$(15%)	22.5	multimodal	0.582±0.077	-21.1±2.2	multimodal	1.786±1.105	-21.3±1.2

3. SEM images of PLGA nanoparticles without mitomycin

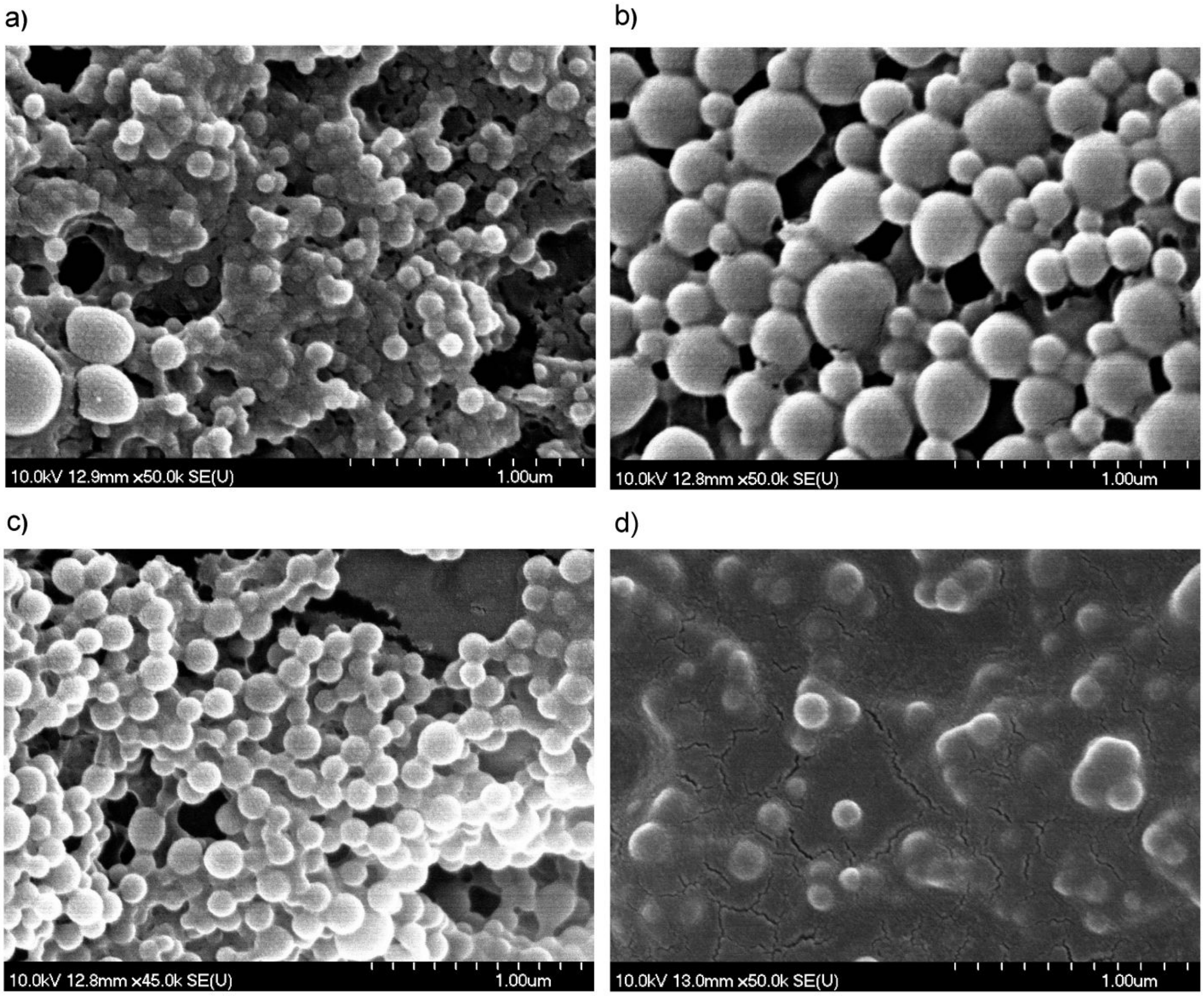

Figure S5. SEM images of empty PLGA nanoparticles stabilized by (a) PSS-MA-g-$C_{16}OH$(15%) (b) PSS-MA-g-$C_{16}NH_2$(15%), (c) PAA-$C_{12}OH$(40%) and (d) PAA-$C_{16}OH$(15%) at the optimal concentrations (see Fig. 1)

5. Macroscopic images

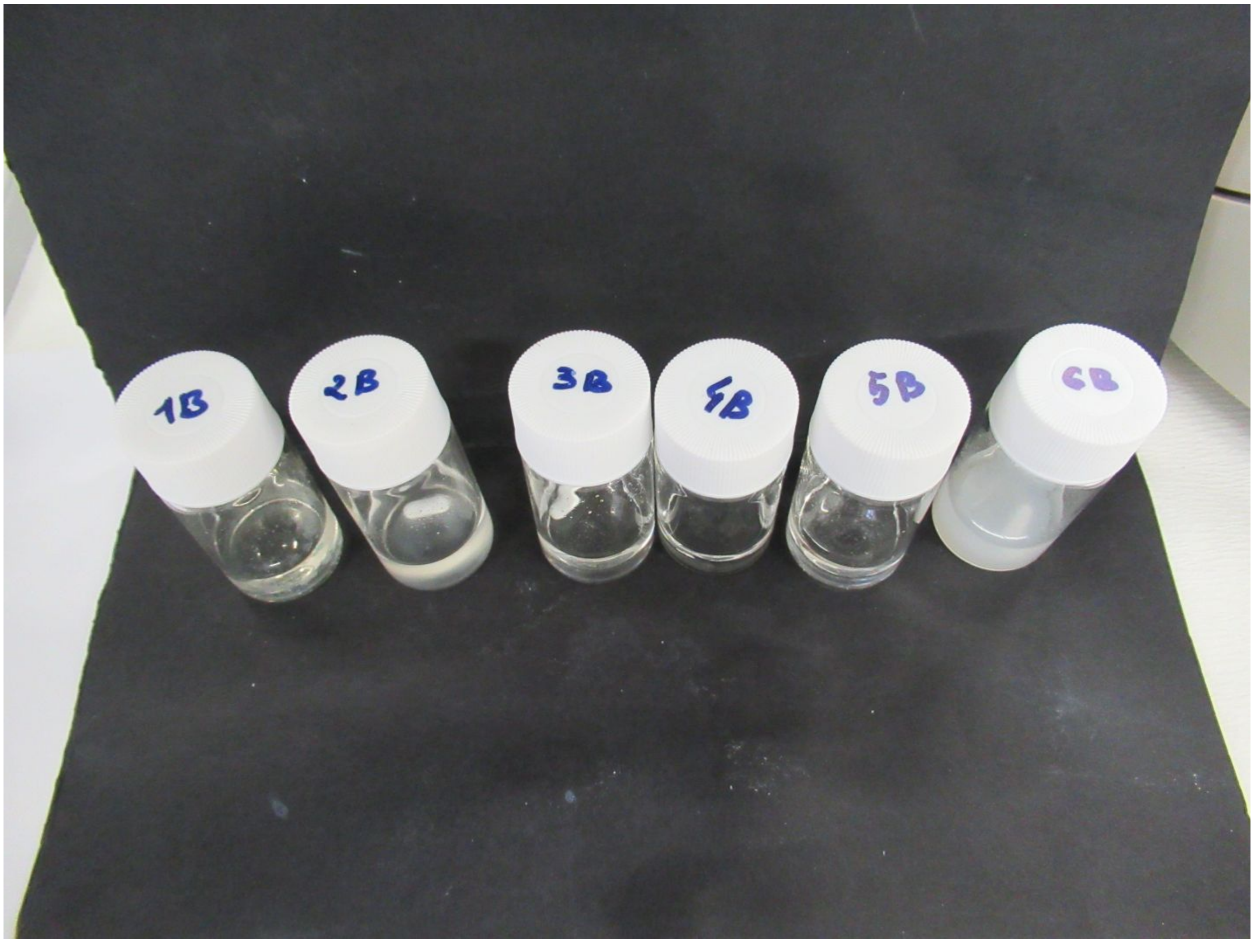

Figure S6. Macroscopic images PLGA dispertsions, stabilized by: (1B) PSS-MA-g-$C_{16}OH$ (15%) at concentration 0.5 mg/mL, (2B) PSS-MA-g-$C_{16}NH_2$ (15%) at concentration 0.5 mg/mL, (3B) PAA-g-$C_{16}OH$ (15%) at concentration 0.5 mg/mL, (4B) PAA-g-$C_{12}OH$ (40%) at concentration 1 mg/mL, (5B) PSS-MA-$C_{16}OH$ (15%) at concentration 0.5 mg/mL (loded with MMC – system 4 according to Table 3, (6B) PSS-MA-g-$C_{16}OH$ (15%) at concentration 22.5 mg/mL.

6. Fitting release data to Korsmeyer-Peppas and Weibull models

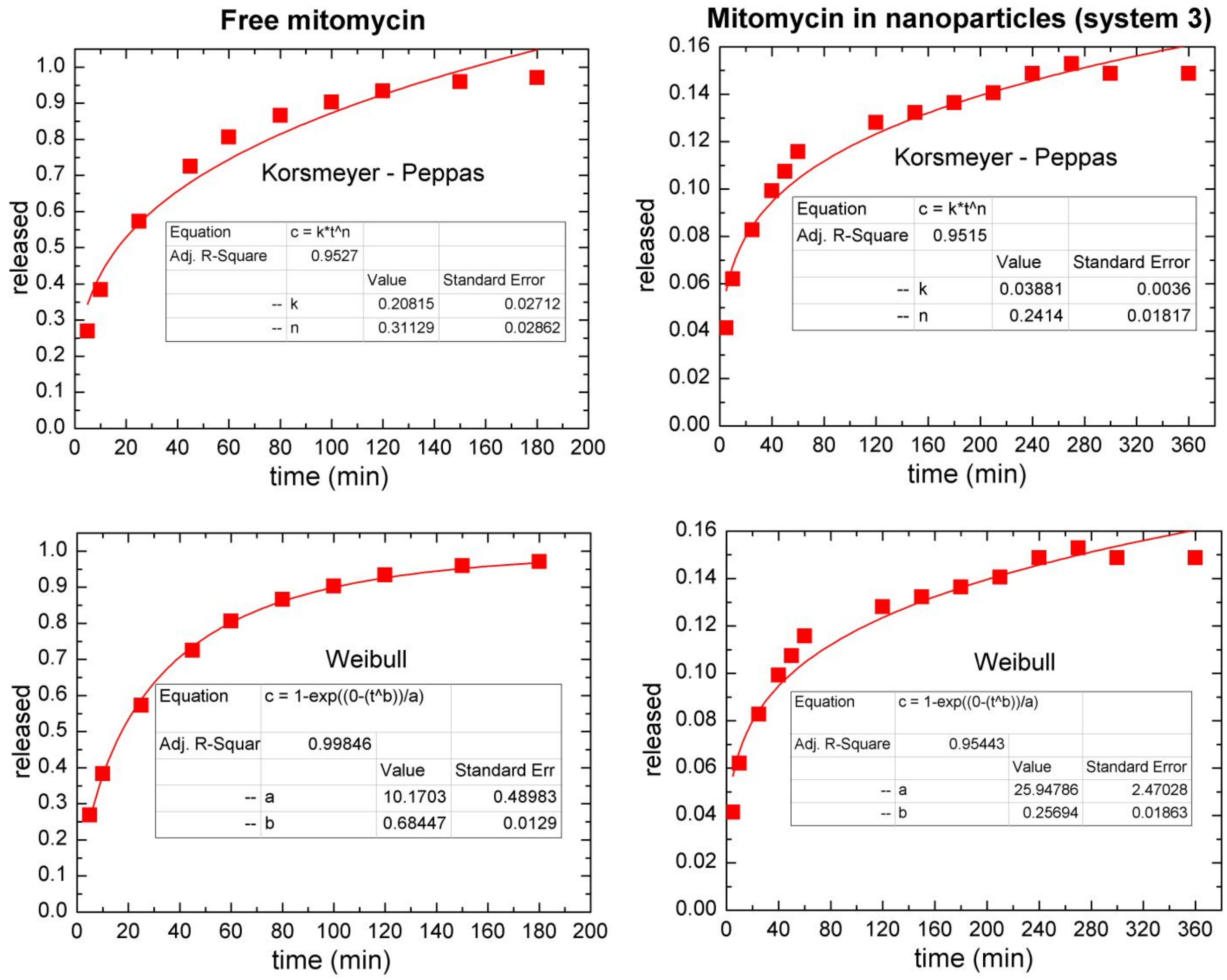

Figure S7. Fitting release data to Korsmeyer-Peppas and Weibull models.
